# Supplementary material for: The Evolution and Characterization of the RNA Interference Pathways in Lophotrochozoa
Source: Genome Biol Evol. 2024 May 7;16(5):evae098. doi: 10.1093/gbe/evae098 (PMC11114477; doi:10.1093/gbe/evae098)
Supplement: evae098_Supplementary_Data [file evae098_supplementary_data.zip › Supplementary_materials.zip!/selected_miRNAs/NoGe_selected_miRNAs/mirdeep_pdf_files/NMRB01000819.1_Notospermus_geniculatus_strain_Ushimado_scaffold819,_whole_genome_shotgun_sequence_21651.pdf]

Provisional ID : NMRB01000819.1_Notospermus_geniculatus_strain_Ushimado_scaffold819,_whole_genome_shotgun_sequence_21651
Score total : 977761.5
Score for star read(s) : 3.9
Score for read counts : 977755.5
Score for mfe : -0.8
Score for randfold : 0
Score for cons. seed : 3
Total read count : 1917837
Mature read count : 1917797
Loop read count : 0
Star read count : 40

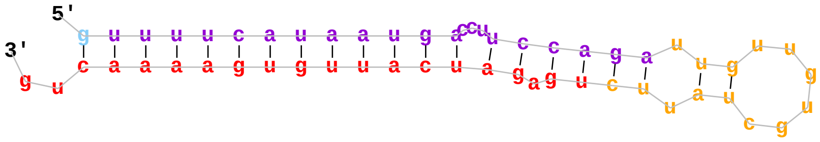

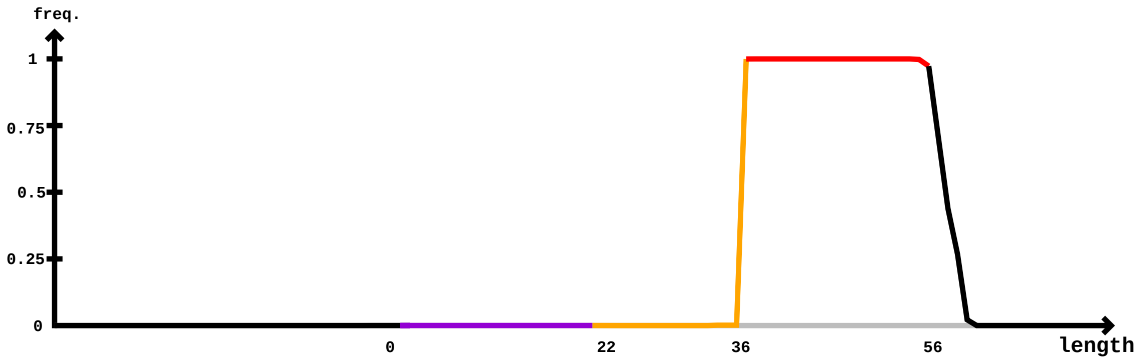

Star Mature

```
5'- ugauguuggcuguaaugaccuucagacgaaacugguuuucauaaugaccuuccagauuguugugcuauucugagaucauugugaaaacugauuccguugccccuaauc  -3'  obs
    ugauguuggcuguaaugaccuucagacgaaacugguuuucauaaugaccuuccagauuguugugcuauucugagaucauugugaaaacugauuccguugccccuaauc       exp
    ....(((((..((((((.....(((......)))(((((((((((((...((((((.((......)).)))).)))))))))))))))......))))))..))))).   reads   mm   sample
    ...............................cugguuuucauaaugaccuuccag.....................................................   2       0    Mm3
    ...............................cugguuuucauaaugaccuuccaga....................................................   3       0    Mm3
    ...................................uuuucauaaugaccuuccaga....................................................   1       0    Mm3
    ...................................uuuucauaaugaccuuAcaga....................................................   1       1    Mm3
    ........................................................uuguugugcuauucugagaucauugug.........................   1       0    Mm3
    ...................................................................uucugagaucauugugaaaacG...................   1       1    Mm3
    ...................................................................uucugagaucGuugugaaaacu...................   1       1    Mm3
    ...................................................................uucugagaucauugugaaaacu...................   63      0    Mm3
    ...................................................................uucugagaucauugugaaaacuU..................   1       1    Mm3
    ....................................................................ucugagaucauugugaaaacug..................   1       0    Mm3
    .....................................................................cugagaucauugugaaaacugauu...............   1       0    Mm3
    ......................................................................ugagaucauugugaaaaA....................   25      1    Mm3
    ......................................................................ugagaucauugugaaaac....................   51      0    Mm3
    ......................................................................ugagaucauugugaaaaU....................   6       1    Mm3
    ......................................................................ugagaucauugugaaaaG....................   12      1    Mm3
    ......................................................................ugUgaucauugugaaaacu...................   2       1    Mm3
    ......................................................................ugagaucauugugGaaacu...................   2       1    Mm3
    ......................................................................ugagaucauugugaGaacu...................   1       1    Mm3
    ......................................................................ugGgaucauugugaaaacu...................   1       1    Mm3
    ......................................................................ugagaucauugugaaGacu...................   3       1    Mm3
    ......................................................................ugagaucauugugaaaaUu...................   7       1    Mm3
    ......................................................................Ngagaucauugugaaaacu...................   1       1    Mm3
    ......................................................................ugagaucauugugaaaacA...................   1       1    Mm3
    ......................................................................ugagaucauugugaaaaGu...................   5       1    Mm3
    ......................................................................ugagaucauugugaaaGcu...................   1       1    Mm3
    ......................................................................ugagaucauugugaaaUcu...................   5       1    Mm3
    ......................................................................ugagGucauugugaaaacu...................   1       1    Mm3
    ......................................................................ugagaucauugugaaaacu...................   1295    0    Mm3
    ......................................................................ugagaucauuCugaaaacu...................   1       1    Mm3
    ......................................................................ugagaucauugugaaaacG...................   2       1    Mm3
    ......................................................................ugagaucGuugugaaaacu...................   4       1    Mm3
    ......................................................................ugagaucauugugaaaaAu...................   16      1    Mm3
    ......................................................................ugagaucauugugaaaCcu...................   3       1    Mm3
```

```
                                   Star                                     Mature
ugauguuggcuguaaugaccuucagacgaaacugguuuucauaaugaccuuccagauuguugugcuauucugagaucauugugaaaacugauuccguugccccuaauc
.....................................................................Cgagaucauugugaaaacu...................   1       1   Mm3
.....................................................................ugagauUauugugaaaacug..................   1       1   Mm3
.....................................................................ugagaucauugugGaaacug..................   1       1   Mm3
.....................................................................ugagauGauugugaaaacug..................   1       1   Mm3
.....................................................................ugagaucauugugaaaCcug..................   19      1   Mm3
.....................................................................ugagaucauuCugaaaacug..................   14      1   Mm3
.....................................................................ugagaucauuAugaaaacug..................   16      1   Mm3
.....................................................................ugagaucauugugaaaacuA..................   52      1   Mm3
.....................................................................ugagaucauugugUaaacug..................   2       1   Mm3
.....................................................................ugagaucauugugaUaacug..................   2       1   Mm3
.....................................................................ugagaucauugugaaaaAug..................   76      1   Mm3
.....................................................................ugagaGcauugugaaaacug..................   1       1   Mm3
.....................................................................ugagaucauugAgaaaacug..................   2       1   Mm3
.....................................................................ugagaucauugugaaaaUug..................   58      1   Mm3
.....................................................................ugagaucGuugugaaaacug..................   4       1   Mm3
.....................................................................ugagaucauugugaaaGcug..................   15      1   Mm3
.....................................................................Agagaucauugugaaaacug..................   2       1   Mm3
.....................................................................Cgagaucauugugaaaacug..................   7       1   Mm3
.....................................................................ugagaucauugugaaUacug..................   2       1   Mm3
.....................................................................ugagUucauugugaaaacug..................   1       1   Mm3
.....................................................................ugaAaucauugugaaaacug..................   1       1   Mm3
.....................................................................Ngagaucauugugaaaacug..................   5       1   Mm3
.....................................................................ugagaucauugugaaaacGg..................   20      1   Mm3
.....................................................................uCagaucauugugaaaacug..................   1       1   Mm3
.....................................................................ugagaucauugugaaaacuU..................   23      1   Mm3
.....................................................................ugagaucauugugaaaUcug..................   12      1   Mm3
.....................................................................ugagCucauugugaaaacug..................   5       1   Mm3
.....................................................................ugagaucauuguAaaaacug..................   2       1   Mm3
.....................................................................ugagaucauugugaaaaGug..................   36      1   Mm3
.....................................................................ugagaucauugugaaaacuC..................   1       1   Mm3
.....................................................................ugUgaucauugugaaaacug..................   1       1   Mm3
.....................................................................ugagaucauugugaaaacAg..................   1       1   Mm3
.....................................................................uAagaucauugugaaaacug..................   7       1   Mm3
.....................................................................ugagaucauugugaaaacCg..................   1       1   Mm3
.....................................................................ugagaucaAugugaaaacug..................   1       1   Mm3
.....................................................................uUagaucauugugaaaacug..................   1       1   Mm3
.....................................................................ugagaucauugugaaGacug..................   4       1   Mm3
.....................................................................uNagaucauugugaaaacug..................   1       1   Mm3
.....................................................................ugCgaucauugugaaaacug..................   1       1   Mm3
.....................................................................Ggagaucauugugaaaacug..................   2       1   Mm3
.....................................................................ugGgaucauugugaaaacug..................   3       1   Mm3
.....................................................................ugagaucauCgugaaaacug..................   3       1   Mm3
.....................................................................ugagaucauuUugaaaacug..................   14      1   Mm3
.....................................................................ugagaAcauugugaaaacug..................   2       1   Mm3
.....................................................................ugagaucauugugaaaacug..................   9259    0   Mm3
.....................................................................Ngagaucauugugaaaacuga.................   5       1   Mm3
.....................................................................ugagaucauugugaGaacuga.................   3       1   Mm3
.....................................................................ugagaucauugCgaaaacuga.................   1       1   Mm3
.....................................................................ugagaucauugugaaaGcuga.................   18      1   Mm3
.....................................................................ugaUaucauugugaaaacuga.................   1       1   Mm3
.....................................................................ugagaucauAgugaaaacuga.................   2       1   Mm3
.....................................................................ugagaucauugugaaUacuga.................   1       1   Mm3
.....................................................................uAagaucauugugaaaacuga.................   5       1   Mm3
.....................................................................ugagaucauuguAaaaacuga.................   3       1   Mm3
.....................................................................ugagaucauugugaaaaUuga.................   87      1   Mm3
.....................................................................ugagaucauuUugaaaacuga.................   13      1   Mm3
.....................................................................ugagaucUuugugaaaacuga.................   9       1   Mm3
.....................................................................ugNgaucauugugaaaacuga.................   1       1   Mm3
.....................................................................Cgagaucauugugaaaacuga.................   5       1   Mm3
.....................................................................ugagCucauugugaaaacuga.................   3       1   Mm3
.....................................................................ugagaucauugugaaaaAuga.................   121     1   Mm3
.....................................................................ugagaNcauugugaaaacuga.................   1       1   Mm3
.....................................................................ugagaucauugugaaaacugC.................   8       1   Mm3
.....................................................................ugagaucauugugaaGacuga.................   6       1   Mm3
.....................................................................ugGgaucauugugaaaacuga.................   2       1   Mm3
.....................................................................ugUgaucauugugaaaacuga.................   3       1   Mm3
.....................................................................ugagaucauugugaaaacuga.................   15493   0   Mm3
.....................................................................ugagaucaCugugaaaacuga.................   2       1   Mm3
.....................................................................ugagaucauugugaUaacuga.................   1       1   Mm3
.....................................................................ugagaucauugugGaaacuga.................   3       1   Mm3
```

```
                                            Star                          Mature
ugauguuggcuguaaugaccuucagacgaaacugguuuucauaaugaccuuccagauuguugugcuauucugagaucauugugaaaacugauuccguugccccuaauc
......................................................................ugagauUauugugaaaacuga.................   5     1   Mm3
......................................................................ugagaucauugugaaaacugG.................   217   1   Mm3
......................................................................ugagaucauuAugaaaacuga.................   24    1   Mm3
......................................................................ugagaucauugugaaaacCga.................   2     1   Mm3
......................................................................ugagaucauugugaaaacugU.................   282   1   Mm3
......................................................................ugagaucGuugugaaaacuga.................   5     1   Mm3
......................................................................ugagaucauuCugaaaacuga.................   14    1   Mm3
......................................................................ugagaCcauugugaaaacuga.................   2     1   Mm3
......................................................................ugCgaucauugugaaaacuga.................   1     1   Mm3
......................................................................ugagaucauugugaaaCcuga.................   22    1   Mm3
......................................................................ugagaucauugugaaaUcuga.................   28    1   Mm3
......................................................................ugagGucauugugaaaacuga.................   1     1   Mm3
......................................................................ugagaucauugugUaaacuga.................   1     1   Mm3
......................................................................ugagaucauugugaaaacuCa.................   2     1   Mm3
......................................................................ugagaAcauugugaaaacuga.................   1     1   Mm3
......................................................................ugagaucauugugaaaaGuga.................   65    1   Mm3
......................................................................uUagaucauugugaaaacuga.................   2     1   Mm3
......................................................................ugagaucauugugaaaacuAa.................   1     1   Mm3
......................................................................ugagaucauuguCaaaacuga.................   3     1   Mm3
......................................................................ugagaucauugugaaaacuUa.................   2     1   Mm3
......................................................................ugagaucauCgugaaaacuga.................   2     1   Mm3
......................................................................ugagaucauugugaaaaAugau................   129   1   Mm3
......................................................................ugagaucauugugaaaacuCau................   2     1   Mm3
......................................................................uNagaucauugugaaaacugau................   1     1   Mm3
......................................................................Cgagaucauugugaaaacugau................   11    1   Mm3
......................................................................ugagGucauugugaaaacugau................   1     1   Mm3
......................................................................ugagaucGuugugaaaacugau................   5     1   Mm3
......................................................................ugagaucauugugaaaaGugau................   70    1   Mm3
......................................................................ugagaucauugugaaCacugau................   1     1   Mm3
......................................................................ugagaucauGgugaaaacugau................   1     1   Mm3
......................................................................ugagaucauugugaaaacCgau................   3     1   Mm3
......................................................................ugagaucauugugaaUacugau................   1     1   Mm3
......................................................................ugCgaucauugugaaaacugau................   2     1   Mm3
......................................................................ugagaucauugugaaaUcugau................   35    1   Mm3
......................................................................ugagaGcauugugaaaacugau................   1     1   Mm3
......................................................................ugagaucauugugGaaacugau................   2     1   Mm3
......................................................................ugagaucauugugaaaCcugau................   22    1   Mm3
......................................................................ugagaucauugugaaaacugUu................   9     1   Mm3
......................................................................ugagaucauugCgaaaacugau................   1     1   Mm3
......................................................................ugagaAcauugugaaaacugau................   1     1   Mm3
......................................................................ugagaucauugugaaaacAgau................   1     1   Mm3
......................................................................ugagaucauugugaaGacugau................   2     1   Mm3
......................................................................ugagaucauuguUaaaacugau................   1     1   Mm3
......................................................................ugagauNauugugaaaacugau................   1     1   Mm3
......................................................................ugagaucauugugaGaacugau................   4     1   Mm3
......................................................................ugUgaucauugugaaaacugau................   6     1   Mm3
......................................................................ugagaucaGugugaaaacugau................   1     1   Mm3
......................................................................ugGgaucauugugaaaacugau................   4     1   Mm3
......................................................................uAagaucauugugaaaacugau................   3     1   Mm3
......................................................................ugagaucauuUugaaaacugau................   12    1   Mm3
......................................................................ugagaucauugugaaaacugaC................   4     1   Mm3
......................................................................ugagaucCuugugaaaacugau................   2     1   Mm3
......................................................................ugaAaucauugugaaaacugau................   1     1   Mm3
......................................................................ugagaucauugugUaaacugau................   1     1   Mm3
......................................................................Ngagaucauugugaaaacugau................   9     1   Mm3
......................................................................ugagaucauugAgaaaacugau................   1     1   Mm3
......................................................................ugagaucauuAugaaaacugau................   27    1   Mm3
......................................................................ugagaucauugugaaaacugaG................   1     1   Mm3
......................................................................ugagaucauuguAaaaacugau................   1     1   Mm3
......................................................................ugagaucauugugaaaacugGu................   3     1   Mm3
......................................................................ugagaucauugugCaaacugau................   3     1   Mm3
......................................................................ugagaucauugugaaaacuUau................   3     1   Mm3
......................................................................ugagaucauugugaaaacugaA................   115   1   Mm3
......................................................................ugagaNcauugugaaaacugau................   1     1   Mm3
......................................................................ugagaucauugugaaaacuAau................   1     1   Mm3
......................................................................ugagaucauugugaaaaUugau................   71    1   Mm3
......................................................................Agagaucauugugaaaacugau................   2     1   Mm3
......................................................................ugagaucauuguCaaaacugau................   1     1   Mm3
......................................................................ugagaucauAgugaaaacugau................   2     1   Mm3
......................................................................ugagaucauuCugaaaacugau................   14    1   Mm3
```

Star Mature

```
ugauguuggcuguaaugaccuucagacgaaacugguuuucauaaugaccuuccagauuguugugcuauucugagaucauugugaaaacugauuccguugccccuaauc
......................................................................ugagCucauugugaaaacugau................	6	1	Mm3
......................................................................ugagauUauugugaaaacugau................	5	1	Mm3
......................................................................ugagaucauugugaaaGcugau................	24	1	Mm3
......................................................................ugagaucauugugaaaacugau................	15605	0	Mm3
......................................................................ugagaucUuugugaaaacugau................	3	1	Mm3
......................................................................ugagaucauugugaaaacugCu................	2	1	Mm3
......................................................................ugagaucauugCgaaaacugauu...............	5	1	Mm3
......................................................................ugagaucaAugugaaaacugauu...............	1	1	Mm3
......................................................................ugagaucauugugaaaacugauG...............	12	1	Mm3
......................................................................ugagGucauugugaaaacugauu...............	4	1	Mm3
......................................................................ugagaucauugugaaaacCgauu...............	4	1	Mm3
......................................................................ugagaucauuCugaaaacugauu...............	16	1	Mm3
......................................................................ugagaucauugGgaaaacugauu...............	1	1	Mm3
......................................................................ugagUucauugugaaaacugauu...............	2	1	Mm3
......................................................................ugagaucauuUugaaaacugauu...............	16	1	Mm3
......................................................................ugagaucauugugaaaacNgauu...............	1	1	Mm3
......................................................................Ngagaucauugugaaaacugauu...............	9	1	Mm3
......................................................................ugagaucCuugugaaaacugauu...............	2	1	Mm3
......................................................................uUagaucauugugaaaacugauu...............	3	1	Mm3
......................................................................ugagaucauugugaaUacugauu...............	2	1	Mm3
......................................................................ugagaucauugugaaaacAgauu...............	2	1	Mm3
......................................................................ugagaucauugugaaaacugaCu...............	5	1	Mm3
......................................................................ugaAaucauugugaaaacugauu...............	1	1	Mm3
......................................................................ugagaAcauugugaaaacugauu...............	1	1	Mm3
......................................................................ugCgaucauugugaaaacugauu...............	2	1	Mm3
......................................................................ugagauUauugugaaaacugauu...............	6	1	Mm3
......................................................................ugagaucauugugaaaacugauC...............	6	1	Mm3
......................................................................uAagaucauugugaaaacugauu...............	5	1	Mm3
......................................................................ugagaucauGgugaaaacugauu...............	1	1	Mm3
......................................................................ugagaucauugugaaaUcugauu...............	43	1	Mm3
......................................................................ugagaNcauugugaaaacugauu...............	4	1	Mm3
......................................................................ugagaucaGugugaaaacugauu...............	1	1	Mm3
......................................................................ugaUaucauugugaaaacugauu...............	1	1	Mm3
......................................................................ugagaucauugugaaaaAugauu...............	149	1	Mm3
......................................................................ugagauNauugugaaaacugauu...............	2	1	Mm3
......................................................................ugagaucauuguAaaaacugauu...............	3	1	Mm3
......................................................................ugagCucauugugaaaacugauu...............	8	1	Mm3
......................................................................ugagaucauAgugaaaacugauu...............	1	1	Mm3
......................................................................ugagaucauugugaaaacugCuu...............	6	1	Mm3
......................................................................Cgagaucauugugaaaacugauu...............	5	1	Mm3
......................................................................ugagaucauugugaaaacugauu...............	18864	0	Mm3
......................................................................ugagaucauugugaaaCcugauu...............	41	1	Mm3
......................................................................ugagaucauugugaUaacugauu...............	1	1	Mm3
......................................................................ugagaucauugugGaaacugauu...............	3	1	Mm3
......................................................................ugagaucauuguCaaaacugauu...............	1	1	Mm3
......................................................................ugagaucauugugaaaacuUauu...............	2	1	Mm3
......................................................................ugagaucauugugaaaaGugauu...............	84	1	Mm3
......................................................................ugagaucauuguUaaaacugauu...............	2	1	Mm3
......................................................................ugagaucaCugugaaaacugauu...............	4	1	Mm3
......................................................................ugagaucauugugaaaacugaAu...............	5	1	Mm3
......................................................................ugUgaucauugugaaaacugauu...............	5	1	Mm3
......................................................................ugagaucauugugaaaacugUuu...............	4	1	Mm3
......................................................................ugagaucauugugaGaacugauu...............	2	1	Mm3
......................................................................ugGgaucauugugaaaacugauu...............	10	1	Mm3
......................................................................ugagaucauugugaaaGcugauu...............	29	1	Mm3
......................................................................ugagaucGuugugaaaacugauu...............	11	1	Mm3
......................................................................ugagaucauugugaaaacugauA...............	158	1	Mm3
......................................................................ugagaucauugugUaaacugauu...............	4	1	Mm3
......................................................................ugagaucauuAugaaaacugauu...............	40	1	Mm3
......................................................................ugagaucauugugaaGacugauu...............	5	1	Mm3
......................................................................Agagaucauugugaaaacugauu...............	2	1	Mm3
......................................................................ugagaucauugugaaaaUugauu...............	93	1	Mm3
......................................................................Ggagaucauugugaaaacugauu...............	2	1	Mm3
......................................................................ugagaucauugugaaaacugaGu...............	1	1	Mm3
......................................................................ugagaucauugugaaaacuAauu...............	1	1	Mm3
......................................................................ugagaucauugugaaaaAugauuc..............	1	1	Mm3
......................................................................ugagaucauugugaaaacugauuU..............	1815	1	Mm3
......................................................................ugagaucauugugaaaacugauuc..............	2	0	Mm3
......................................................................ugagaucauugugaaaacugauuA..............	62	1	Mm3
......................................................................ugagaucauugugaaaCcugauuc..............	1	1	Mm3
```

```
                                            Star                                Mature
ugauguuggcuguaaugaccuucagacgaaacugguuuucauaaugaccuuccagauuguugugcuauucugagaucauugugaaaacugauuccguugccccuaauc
......................................................................ugagaucauugugaaaacugauuG..............    1      1     Mm3
.......................................................................gagaucauugugaaaacu...................    1      0     Mm3
.......................................................................gagaucauugugaaaacug..................    8      0     Mm3
.......................................................................gagaucauugCgaaaacuga.................    1      1     Mm3
.......................................................................gagaucauugugaaaacuga.................    1      0     Mm3
.......................................................................gagaucauugugaaaacugau................    1      0     Mm3
.......................................................................gagaucauugugaaaacugauu...............    3      0     Mm3
.......................................................................gagaAcauugugaaaacugauu...............    1      1     Mm3

...............................cugguuuucauaaugaccuucc.......................................................    4      0     Mm1
...............................cugguuuucauaaugaUcuuccag.....................................................    1      1     Mm1
...............................cugguuuucauaaugaccuuccag.....................................................    12     0     Mm1
...................................uuuucauaaugaccuuccaga....................................................    1      0     Mm1
....................................uuucauaaugaccuuccaga....................................................    2      0     Mm1
..................................................................auucugagaucauugugUaaa.....................    1      1     Mm1
..................................................................auucugagaucauugugaaaa.....................    7      0     Mm1
...................................................................uucugagaucauugugaCaacu...................    2      1     Mm1
...................................................................uucugagaucauugugaaaacu...................    126    0     Mm1
...................................................................uucugagaucauugugaUaacu...................    1      1     Mm1
...................................................................uucugagaucauugugCaaacu...................    1      1     Mm1
...................................................................uucugagaucauuguUaaaacu...................    1      1     Mm1
...................................................................uucugagaucauugugUaaacu...................    1      1     Mm1
...................................................................uucugagaucauugugaaaacuA..................    5      1     Mm1
.....................................................................cugagaucauugugaaaacug..................    6      0     Mm1
......................................................................ugagaucauugugaaaGc....................    2      1     Mm1
......................................................................ugagaucauugugaaaaU....................    2      1     Mm1
......................................................................ugagaucauugugaaaaG....................    45     1     Mm1
......................................................................ugagaucauugugaaaac....................    71     0     Mm1
......................................................................ugagaucauugugaaaaA....................    42     1     Mm1
......................................................................ugagaucUuugugaaaacu...................    1      1     Mm1
......................................................................ugagaucauugugaaaGcu...................    2      1     Mm1
......................................................................Ngagaucauugugaaaacu...................    3      1     Mm1
......................................................................uAagaucauugugaaaacu...................    1      1     Mm1
......................................................................ugagaucauuCugaaaacu...................    1      1     Mm1
......................................................................ugagaucauugugaaaaUu...................    14     1     Mm1
......................................................................ugagaucauugugaaaUcu...................    5      1     Mm1
......................................................................ugagaucauugugaaaaAu...................    28     1     Mm1
......................................................................ugagaucauuUugaaaacu...................    2      1     Mm1
......................................................................ugagaAcauugugaaaacu...................    1      1     Mm1
......................................................................ugagaucGuugugaaaacu...................    4      1     Mm1
......................................................................ugagaucauugugaaaCcu...................    5      1     Mm1
......................................................................ugagCucauugugaaaacu...................    2      1     Mm1
......................................................................ugGgaucauugugaaaacu...................    1      1     Mm1
......................................................................ugUgaucauugugaaaacu...................    3      1     Mm1
......................................................................ugagaucauugugaaaaGu...................    17     1     Mm1
......................................................................Cgagaucauugugaaaacu...................    1      1     Mm1
......................................................................ugagaucauugugaaaacu...................    2414   0     Mm1
......................................................................ugCgaucauugugaaaacu...................    1      1     Mm1
......................................................................ugagaucauGgugaaaacu...................    1      1     Mm1
......................................................................ugagaucauugugGaaacug..................    6      1     Mm1
......................................................................ugagaucauugugaaCacug..................    1      1     Mm1
......................................................................Ggagaucauugugaaaacug..................    1      1     Mm1
......................................................................ugagUucauugugaaaacug..................    3      1     Mm1
......................................................................uAagaucauugugaaaacug..................    11     1     Mm1
......................................................................ugagaucauAgugaaaacug..................    2      1     Mm1
......................................................................uCagaucauugugaaaacug..................    2      1     Mm1
......................................................................ugagaucauugugaUaacug..................    2      1     Mm1
......................................................................ugagaucauugugaaaaGug..................    122    1     Mm1
......................................................................ugagaucUuugugaaaacug..................    3      1     Mm1
......................................................................ugagauUauugugaaaacug..................    6      1     Mm1
......................................................................ugUgaucauugugaaaacug..................    6      1     Mm1
......................................................................ugaAaucauugugaaaacug..................    2      1     Mm1
......................................................................Ngagaucauugugaaaacug..................    18     1     Mm1
......................................................................ugagaucauugugaaUacug..................    3      1     Mm1
......................................................................ugagaucauugugaaaaUug..................    152    1     Mm1
......................................................................ugaUaucauugugaaaacug..................    1      1     Mm1
......................................................................ugagaucauugugaaaacuA..................    44     1     Mm1
......................................................................ugagauGauugugaaaacug..................    1      1     Mm1
......................................................................ugGgaucauugugaaaacug..................    18     1     Mm1
......................................................................ugagaucauugugUaaacug..................    4      1     Mm1
```

Star Mature

ugauguuggcuguaaugaccuucagacgaaacugguuuucauaaugaccuuccagauuguugugcuauucugagaucauugugaaaacugauuccguugccccuaauc			
....ugagaGcauugugaaaacug..................	2	1	Mm1
....ugagaAcauugugaaaacug..................	1	1	Mm1
....Agagaucauugugaaaacug..................	2	1	Mm1
....ugagaucauugCgaaaacug..................	4	1	Mm1
....ugagaucauugugaaaCcug..................	58	1	Mm1
....ugagaucauGgugaaaacug..................	5	1	Mm1
....ugagaucauugGgaaaacug..................	1	1	Mm1
....ugagaucauugugaaaacug..................	29250	0	Mm1
....ugagaucauugugaaGacug..................	4	1	Mm1
....ugagaucaCugugaaaacug..................	3	1	Mm1
....ugagaucauuguAaaaacug..................	9	1	Mm1
....ugagaucauuUugaaaacug..................	30	1	Mm1
....ugagaucauugugaaaacuC..................	1	1	Mm1
....ugagaucaAugugaaaacug..................	1	1	Mm1
....ugagGucauugugaaaacug..................	2	1	Mm1
....ugagaNcauugugaaaacug..................	7	1	Mm1
....ugagaucaGugugaaaacug..................	3	1	Mm1
....Cgagaucauugugaaaacug..................	6	1	Mm1
....ugagaucauugugaGaacug..................	3	1	Mm1
....ugagaucauuAugaaaacug..................	80	1	Mm1
....ugagauAauugugaaaacug..................	4	1	Mm1
....ugagaucauuCugaaaacug..................	22	1	Mm1
....ugagaucauugugaaaaAug..................	272	1	Mm1
....ugagaCcauugugaaaacug..................	1	1	Mm1
....ugagaucauugugaaaUcug..................	57	1	Mm1
....ugagCucauugugaaaacug..................	13	1	Mm1
....ugagaucauCgugaaaacug..................	6	1	Mm1
....uUagaucauugugaaaacug..................	7	1	Mm1
....ugagaucauuguCaaaacug..................	1	1	Mm1
....ugagaucauugugaaaacuU..................	27	1	Mm1
....ugagaucauugAgaaaacug..................	2	1	Mm1
....ugagaucauugugaaaacCg..................	1	1	Mm1
....ugagaucauuguUaaaacug..................	4	1	Mm1
....ugagaucauugugaaaacGg..................	14	1	Mm1
....ugagaucGuugugaaaacug..................	17	1	Mm1
....ugagaucauugugaaaGcug..................	35	1	Mm1
....ugagaucauugugaaaacugU.................	267	1	Mm1
....ugagaucauugugGaaacuga.................	1	1	Mm1
....ugagaucauugugaaaacuga.................	12838	0	Mm1
....ugaAaucauugugaaaacuga.................	1	1	Mm1
....ugagaucauugugaaaacuCa.................	2	1	Mm1
....ugagaucauugugaaaaGuga.................	51	1	Mm1
....ugagaucaCugugaaaacuga.................	4	1	Mm1
....ugagaGcauugugaaaacuga.................	1	1	Mm1
....ugGgaucauugugaaaacuga.................	5	1	Mm1
....ugagaucCuugugaaaacuga.................	2	1	Mm1
....ugagaNcauugugaaaacuga.................	1	1	Mm1
....ugagaucauuCugaaaacuga.................	17	1	Mm1
....ugUgaucauugugaaaacuga.................	3	1	Mm1
....uCagaucauugugaaaacuga.................	2	1	Mm1
....ugagaucauugugaGaacuga.................	1	1	Mm1
....ugagaucauuguAaaaacuga.................	2	1	Mm1
....Agagaucauugugaaaacuga.................	1	1	Mm1
....ugagauUauugugaaaacuga.................	2	1	Mm1
....ugagaucauugugaCaacuga.................	1	1	Mm1
....ugagUucauugugaaaacuga.................	2	1	Mm1
....uAagaucauugugaaaacuga.................	9	1	Mm1
....ugagaucauugugUaaacuga.................	3	1	Mm1
....ugaUaucauugugaaaacuga.................	1	1	Mm1
....ugagaucGuugugaaaacuga.................	1	1	Mm1
....ugagaucauCgugaaaacuga.................	2	1	Mm1
....uNagaucauugugaaaacuga.................	1	1	Mm1
....ugagaucauuUugaaaacuga.................	5	1	Mm1
....ugagaucauugugaaaacuAa.................	1	1	Mm1
....ugagaucauugugaaaaUuga.................	61	1	Mm1
....ugagaAcauugugaaaacuga.................	3	1	Mm1
....ugagauGauugugaaaacuga.................	1	1	Mm1
....ugagaucauugugaaaacugG.................	39	1	Mm1
....ugagGucauugugaaaacuga.................	3	1	Mm1
....ugagaucauugugaaaGcuga.................	13	1	Mm1

```
                                        Star                              Mature
ugauguuggcuguaaugaccuucagacgaaacugguuuucauaaugaccuuccagauuguugugcuauucugagaucauugugaaaacugauuccguugccccuaauc
......................................................................ugagaucauugugaaaUcuga.................   27     1    Mm1
......................................................................Ngagaucauugugaaaacuga.................   9      1    Mm1
......................................................................ugagaucauugugaaaCcuga.................   17     1    Mm1
......................................................................ugagaucauugCgaaaacuga.................   7      1    Mm1
......................................................................ugagaucauugugaaGacuga.................   1      1    Mm1
......................................................................ugCgaucauugugaaaacuga.................   1      1    Mm1
......................................................................ugagaucauAgugaaaacuga.................   1      1    Mm1
......................................................................ugagaucauugugCaaacuga.................   1      1    Mm1
......................................................................ugagaucauuAugaaaacuga.................   22     1    Mm1
......................................................................ugagCucauugugaaaacuga.................   5      1    Mm1
......................................................................ugagaucauugugaaaacCga.................   1      1    Mm1
......................................................................ugagaucauugugaaaacugC.................   1      1    Mm1
......................................................................ugagaucauugAgaaaacuga.................   1      1    Mm1
......................................................................ugagaucauugugaaaaAuga.................   103    1    Mm1
......................................................................Cgagaucauugugaaaacuga.................   5      1    Mm1
......................................................................ugagaucGuugugaaaacugau................   6      1    Mm1
......................................................................ugagauUauugugaaaacugau................   2      1    Mm1
......................................................................ugagaucauugugaaaacugCu................   1      1    Mm1
......................................................................ugagaNcauugugaaaacugau................   3      1    Mm1
......................................................................ugagaucaCugugaaaacugau................   1      1    Mm1
......................................................................ugagaucaGugugaaaacugau................   1      1    Mm1
......................................................................ugagaucauugugaaaaUugau................   53     1    Mm1
......................................................................ugagaucauugugaaaacugaA................   117    1    Mm1
......................................................................ugagCucauugugaaaacugau................   3      1    Mm1
......................................................................ugUgaucauugugaaaacugau................   1      1    Mm1
......................................................................ugagaucauugugaaaacugUu................   6      1    Mm1
......................................................................ugagaucauugugUaaacugau................   2      1    Mm1
......................................................................uAagaucauugugaaaacugau................   1      1    Mm1
......................................................................ugagaucauugugaaaaGugau................   32     1    Mm1
......................................................................ugagaucauugugaaaGcugau................   12     1    Mm1
......................................................................ugagaCcauugugaaaacugau................   1      1    Mm1
......................................................................uCagaucauugugaaaacugau................   2      1    Mm1
......................................................................ugagaucCuugugaaaacugau................   1      1    Mm1
......................................................................ugagaucauugugaGaacugau................   2      1    Mm1
......................................................................ugCgaucauugugaaaacugau................   1      1    Mm1
......................................................................ugagaucUuugugaaaacugau................   1      1    Mm1
......................................................................ugagaucauAgugaaaacugau................   1      1    Mm1
......................................................................ugagaucauugugaUaacugau................   1      1    Mm1
......................................................................ugagaucauuCugaaaacugau................   7      1    Mm1
......................................................................ugagaucauuUugaaaacugau................   8      1    Mm1
......................................................................ugagaucauugugaaaacCgau................   1      1    Mm1
......................................................................ugagaucauugugaaGacugau................   1      1    Mm1
......................................................................ugagaucauugugaaaacugau................   9187   0    Mm1
......................................................................ugGgaucauugugaaaacugau................   6      1    Mm1
......................................................................ugagaucauugugaaaCcugau................   17     1    Mm1
......................................................................ugagaucauugugaaaaAugau................   80     1    Mm1
......................................................................ugagaucauugugCaaacugau................   1      1    Mm1
......................................................................ugagaucauugugaaaUcugau................   10     1    Mm1
......................................................................ugagaucauCgugaaaacugau................   9      1    Mm1
......................................................................Ngagaucauugugaaaacugau................   3      1    Mm1
......................................................................ugagaucauugugGaaacugau................   1      1    Mm1
......................................................................ugagaucauugCgaaaacugau................   2      1    Mm1
......................................................................ugagaucauugugaaaacuUau................   4      1    Mm1
......................................................................ugagaucauuAugaaaacugau................   21     1    Mm1
......................................................................ugagaucauugugaaaCcugauu...............   18     1    Mm1
......................................................................ugagaucauugugaaaacugauu...............   9341   0    Mm1
......................................................................ugagaucauugugaaaacugauC...............   4      1    Mm1
......................................................................ugagaucauugugUaaacugauu...............   5      1    Mm1
......................................................................ugaAaucauugugaaaacugauu...............   2      1    Mm1
......................................................................ugGgaucauugugaaaacugauu...............   10     1    Mm1
......................................................................ugagaucauugugaaGacugauu...............   3      1    Mm1
......................................................................ugagaAcauugugaaaacugauu...............   2      1    Mm1
......................................................................uCagaucauugugaaaacugauu...............   1      1    Mm1
......................................................................ugagaucauugugaaaaAugauu...............   71     1    Mm1
......................................................................ugagaNcauugugaaaacugauu...............   2      1    Mm1
......................................................................ugagaucauugugaaaacugGuu...............   2      1    Mm1
......................................................................ugagaucauugugaaaacugauA...............   57     1    Mm1
......................................................................ugagaucauuAugaaaacugauu...............   15     1    Mm1
......................................................................ugagaucauGgugaaaacugauu...............   1      1    Mm1
......................................................................ugagaucaGugugaaaacugauu...............   2      1    Mm1
```

```
                                   Star                                  Mature
ugauguuggcuguaaugaccuucagacgaaacugguuuucauaaugaccuuccagauuguugugcuauucugagaucauugugaaaacugauuccguugccccuaauc
......................................................................ugagaucauugugaaaUcugauu...............  17   1  Mm1
......................................................................ugagaucauugugaaaGcugauu...............  13   1  Mm1
......................................................................ugagaucauugugaaaacugaAu...............  10   1  Mm1
......................................................................ugagaucauugugaaaaUugauu...............  49   1  Mm1
......................................................................ugagaucauuCugaaaacugauu...............  7    1  Mm1
......................................................................ugagauUauugugaaaacugauu...............  1    1  Mm1
......................................................................ugagaucauugugaaaacugUuu...............  2    1  Mm1
......................................................................ugagaucauCgugaaaacugauu...............  1    1  Mm1
......................................................................ugagaucauugugaaaacAgauu...............  2    1  Mm1
......................................................................ugagaucauugugGaaacugauu...............  1    1  Mm1
......................................................................ugagaucauuUugaaaacugauu...............  6    1  Mm1
......................................................................ugagaucauugugaGaacugauu...............  1    1  Mm1
......................................................................ugagGucauugugaaaacugauu...............  1    1  Mm1
......................................................................ugagaucauugCgaaaacugauu...............  1    1  Mm1
......................................................................ugagaucauugugaaaaGugauu...............  43   1  Mm1
......................................................................uAagaucauugugaaaacugauu...............  2    1  Mm1
......................................................................ugagaucUuugugaaaacugauu...............  2    1  Mm1
......................................................................ugagaucGuugugaaaacugauu...............  33   1  Mm1
......................................................................Ngagaucauugugaaaacugauu...............  7    1  Mm1
......................................................................ugagCucauugugaaaacugauu...............  7    1  Mm1
......................................................................ugUgaucauugugaaaacugauu...............  1    1  Mm1
......................................................................ugagaucauugugaaaacugauuG..............  3    1  Mm1
......................................................................ugagaucauugugaaaacugauuU..............  813  1  Mm1
......................................................................ugagaucauugugaaaacugauuA..............  41   1  Mm1
......................................................................ugagaucauugugaaaacugauuc..............  2    0  Mm1
.......................................................................gagaucauugugaaaacuU..................  1    1  Mm1
.......................................................................gagaucauugugaaaacug..................  27   0  Mm1
.......................................................................gagaucauAgugaaaacug..................  1    1  Mm1
.......................................................................gagaucauugugaaaacugau................  1    0  Mm1
.......................................................................gagaucauugugaaaacugauu...............  2    0  Mm1
........................................................................Ugaucauugugaaaacug..................  1    1  Mm1

...............................cugguuuucauaaugaccuucc.......................................................  1    0  Mm2
...............................cugguuuucauaaugaccuCcca......................................................  1    1  Mm2
...............................cugguuuucauaaugacAuuccag.....................................................  1    1  Mm2
...............................cugguuuucauaaugaccuuccag.....................................................  3    0  Mm2
...............................cugguuuucauaaugaccuuccagU....................................................  3    1  Mm2
...............................cugguuuucauaaugaUcuuccaga....................................................  1    1  Mm2
...............................cugguuuucauaaugacUuuccaga....................................................  1    1  Mm2
...............................cugguuuucauaaugaccuuccaga....................................................  29   0  Mm2
...............................cugguuuucauaaugaccuuGcaga....................................................  1    1  Mm2
...................................uuuucauaaugaccuuccag.....................................................  1    0  Mm2
...................................uuuucauaaugaccuuccaga....................................................  10   0  Mm2
.....................................uucauaaugaccuuccaga....................................................  1    0  Mm2
...................................................................uucAgagaucauugugaaaacu...................  1    1  Mm2
...................................................................uucugagaucauugugCaaacu...................  2    1  Mm2
...................................................................uucugagaucauugugUaaacu...................  1    1  Mm2
...................................................................uucugagaucauugugaaaacG...................  1    1  Mm2
...................................................................uucugagaucauugugaCaacu...................  1    1  Mm2
...................................................................uucugagaucauugugaaaacu...................  302  0  Mm2
...................................................................uucugagaucauugugaGaacu...................  2    1  Mm2
...................................................................uucugagaucauugugaaaacug..................  12   0  Mm2
...................................................................uucugagaucauugugaaaacuA..................  2    1  Mm2
...................................................................uucugagaucauugugaaaacuU..................  2    1  Mm2
.....................................................................cugagaucauugugaaaacugau................  1    0  Mm2
.....................................................................cugagaucauugugaaaacugauu...............  3    0  Mm2
......................................................................ugagaucauugugaaaU.....................  3    1  Mm2
......................................................................ugagaucauugugaaaG.....................  37   1  Mm2
......................................................................ugagaucauugugaaac.....................  84   0  Mm2
......................................................................ugagaucGuugugaaac.....................  2    1  Mm2
......................................................................ugagaucauugugaaaA.....................  36   1  Mm2
......................................................................uAagaucauugugaaaacu...................  1    1  Mm2
......................................................................ugagaucGuugugaaaacu...................  7    1  Mm2
......................................................................ugagaucauugugaaaCcu...................  2    1  Mm2
......................................................................ugagaucauugugaaaUcu...................  5    1  Mm2
......................................................................Cgagaucauugugaaaacu...................  1    1  Mm2
......................................................................ugagaucUuugugaaaacu...................  1    1  Mm2
......................................................................ugagauUauugugaaaacu...................  1    1  Mm2
......................................................................ugagaucauuCugaaaacu...................  2    1  Mm2
......................................................................ugagaucauugugaaaacG...................  20   1  Mm2
```

```
                                           Star                                     Mature
ugauguuggcuguaaugaccuucagacgaaacugguuuucauaaugaccuucccagauuguugugcuauucugagaucauugugaaaacugauuccguugccccuaauc
.......................................................................Ngagaucauugugaaaacu...................   3      1   Mm2
.......................................................................ugagaucauCgugaaaacu...................   1      1   Mm2
.......................................................................ugUgaucauugugaaaacu...................   1      1   Mm2
.......................................................................ugagaucauugugaaGacu...................   1      1   Mm2
.......................................................................ugagaucauugugaaaacA...................   1      1   Mm2
.......................................................................ugagaucauugAgaaaacu...................   2      1   Mm2
.......................................................................ugagaCcauugugaaaacu...................   1      1   Mm2
.......................................................................ugagaucauugugaaaaAu...................   29     1   Mm2
.......................................................................ugagaucauugugaaaGcu...................   5      1   Mm2
.......................................................................ugagaucauugugaaaacu...................   2434   0   Mm2
.......................................................................ugagaucauugugaaaaUu...................   15     1   Mm2
.......................................................................ugagaucauuUugaaaacu...................   3      1   Mm2
.......................................................................ugagaucauugugaaaaGu...................   8      1   Mm2
.......................................................................ugagaucaAugugaaaacug..................   1      1   Mm2
.......................................................................ugagaucauCgugaaaacug..................   3      1   Mm2
.......................................................................ugagaucUuugugaaaacug..................   5      1   Mm2
.......................................................................ugagaucauugGgaaaacug..................   1      1   Mm2
.......................................................................ugagaucauugugGaaacug..................   2      1   Mm2
.......................................................................ugagaucauugugaUaacug..................   1      1   Mm2
.......................................................................ugagaucauugugaGaacug..................   2      1   Mm2
.......................................................................Cgagaucauugugaaaacug..................   7      1   Mm2
.......................................................................ugUgaucauugugaaaacug..................   3      1   Mm2
.......................................................................ugagaucauuguAaaaacug..................   6      1   Mm2
.......................................................................ugagaucauugugaaaGcug..................   30     1   Mm2
.......................................................................ugagaucauugAgaaaacug..................   1      1   Mm2
.......................................................................ugagaucaCugugaaaacug..................   5      1   Mm2
.......................................................................ugagaucGuugugaaaacug..................   10     1   Mm2
.......................................................................ugagaucauuUugaaaacug..................   17     1   Mm2
.......................................................................uAagaucauugugaaaacug..................   7      1   Mm2
.......................................................................ugagauAauugugaaaacug..................   2      1   Mm2
.......................................................................ugagaucauugugaaaacuA..................   106    1   Mm2
.......................................................................ugagGucauugugaaaacug..................   3      1   Mm2
.......................................................................ugagaucauugugaaaacuC..................   5      1   Mm2
.......................................................................ugagaucauugCgaaaacug..................   5      1   Mm2
.......................................................................ugagaucauugugaaaCcug..................   53     1   Mm2
.......................................................................ugagaucauugugaaaacGg..................   156    1   Mm2
.......................................................................ugagaCcauugugaaaacug..................   3      1   Mm2
.......................................................................ugagaucauugugaaaaAug..................   224    1   Mm2
.......................................................................ugagaucCuugugaaaacug..................   2      1   Mm2
.......................................................................ugagaucauuCugaaaacug..................   22     1   Mm2
.......................................................................Ngagaucauugugaaaacug..................   12     1   Mm2
.......................................................................ugagaucauugugaCaacug..................   1      1   Mm2
.......................................................................ugGgaucauugugaaaacug..................   9      1   Mm2
.......................................................................ugagauNauugugaaaacug..................   1      1   Mm2
.......................................................................ugagaucauugugaaaacug..................   27979  0   Mm2
.......................................................................ugagaucauugugUaaacug..................   6      1   Mm2
.......................................................................Ggagaucauugugaaaacug..................   1      1   Mm2
.......................................................................ugagaucauuAugaaaacug..................   57     1   Mm2
.......................................................................ugaAaucauugugaaaacug..................   1      1   Mm2
.......................................................................ugagCucauugugaaaacug..................   2      1   Mm2
.......................................................................ugagaucauugugaaaacCg..................   4      1   Mm2
.......................................................................ugagaucauugugaaaUcug..................   44     1   Mm2
.......................................................................ugagaNcauugugaaaacug..................   1      1   Mm2
.......................................................................ugagaucauugugaaUacug..................   2      1   Mm2
.......................................................................ugaUaucauugugaaaacug..................   2      1   Mm2
.......................................................................ugagauUauugugaaaacug..................   9      1   Mm2
.......................................................................ugagaucauugugaaaaGug..................   119    1   Mm2
.......................................................................ugagUucauugugaaaacug..................   3      1   Mm2
.......................................................................ugagaucauuguCaaaacug..................   3      1   Mm2
.......................................................................ugagaAcauugugaaaacug..................   5      1   Mm2
.......................................................................ugagaucauugugaaGacug..................   1      1   Mm2
.......................................................................ugaCaucauugugaaaacug..................   1      1   Mm2
.......................................................................ugagaucauugugaaaacuU..................   69     1   Mm2
.......................................................................uUagaucauugugaaaacug..................   7      1   Mm2
.......................................................................ugagaucauugugCaaacug..................   1      1   Mm2
.......................................................................ugCgaucauugugaaaacug..................   1      1   Mm2
.......................................................................ugagaucauugugaaaaUug..................   125    1   Mm2
.......................................................................ugagaucauuguUaaaacug..................   3      1   Mm2
.......................................................................Agagaucauugugaaaacug..................   4      1   Mm2
.......................................................................ugagaucauuCugaaaacuga.................   18     1   Mm2
```

Star Mature

ugauguuggcuguaaugaccuucagacgaaacugguuuucauaaugaccuuccagauuguugugcuauucugagaucauugugaaaacugaauuccguugccccuaauc			
....ugagaucaGugugaaaacuga..................	3	1	Mm2
....ugagaucauugugaaaacugC..................	9	1	Mm2
....ugagaNcauugugaaaacuga..................	3	1	Mm2
....ugagaucauugugaaaaUuga..................	112	1	Mm2
....Ggagaucauugugaaaacuga..................	1	1	Mm2
....ugagaucauugAgaaaacuga..................	1	1	Mm2
....ugaUaucauugugaaaacuga..................	2	1	Mm2
....ugagaucauugugaaaaAuga..................	210	1	Mm2
....ugagaucCuugugaaaacuga..................	1	1	Mm2
....ugagaucauugugaaaacugU..................	393	1	Mm2
....ugagaucauugugUaaacuga..................	3	1	Mm2
....ugagaucauCgugaaaacuga..................	2	1	Mm2
....ugagaAcauugugaaaacuga..................	4	1	Mm2
....ugagUucauugugaaaacuga..................	9	1	Mm2
....ugagaucauugugaaaCcuga..................	41	1	Mm2
....ugagauNauugugaaaacuga..................	2	1	Mm2
....ugagaucauAgugaaaacuga..................	2	1	Mm2
....ugagaucauugugaaaaGuga..................	131	1	Mm2
....ugagaucauugugaGaacuga..................	3	1	Mm2
....ugagaucauugugCaaacuga..................	1	1	Mm2
....ugagaucauugugaaaacCga..................	2	1	Mm2
....ugaCaucauugugaaaacuga..................	1	1	Mm2
....ugGgaucauugugaaaacuga..................	4	1	Mm2
....ugagGucauugugaaaacuga..................	3	1	Mm2
....ugagaucauugugaaaacugG..................	676	1	Mm2
....ugagaCcauugugaaaacuga..................	3	1	Mm2
....ugagaucauugugaaaUcuga..................	44	1	Mm2
....ugUgaucauugugaaaacuga..................	1	1	Mm2
....Ngagaucauugugaaaacuga..................	7	1	Mm2
....ugagaucauuAugaaaacuga..................	47	1	Mm2
....ugagCucauugugaaaacuga..................	2	1	Mm2
....ugagaucUuugugaaaacuga..................	4	1	Mm2
....ugagaucaAugugaaaacuga..................	2	1	Mm2
....uAagaucauugugaaaacuga..................	9	1	Mm2
....ugagauUauugugaaaacuga..................	8	1	Mm2
....ugagauGauugugaaaacuga..................	1	1	Mm2
....ugagaucauuguUaaaacuga..................	2	1	Mm2
....ugagaucauugugaaCacuga..................	1	1	Mm2
....ugagaucauugugaaaacuga..................	24072	0	Mm2
....ugagaucauugCgaaaacuga..................	4	1	Mm2
....ugagaucaCugugaaaacuga..................	1	1	Mm2
....ugagaucauuUugaaaacuga..................	17	1	Mm2
....ugagaucauugugaaaacuAa..................	6	1	Mm2
....ugagaucauugugaaaGcuga..................	28	1	Mm2
....ugagaucauugugaaGacuga..................	3	1	Mm2
....Cgagaucauugugaaaacuga..................	7	1	Mm2
....Agagaucauugugaaaacuga..................	3	1	Mm2
....ugagaucauugugaaUacuga..................	4	1	Mm2
....ugagaucGuugugaaaacuga..................	23	1	Mm2
....ugaAaucauugugaaaacuga..................	4	1	Mm2
....ugagaGcauugugaaaacuga..................	1	1	Mm2
....uUagaucauugugaaaacuga..................	6	1	Mm2
....ugagaucauugugGaaacuga..................	5	1	Mm2
....ugagaucauugugaaaacuUa..................	4	1	Mm2
....ugaAaucauugugaaaacugau.................	3	1	Mm2
....ugagaAcauugugaaaacugau.................	1	1	Mm2
....ugagaucauuCugaaaacugau.................	36	1	Mm2
....ugagaucauCgugaaaacugau.................	3	1	Mm2
....ugagaucauuguUaaaacugau.................	5	1	Mm2
....uCagaucauugugaaaacugau.................	1	1	Mm2
....ugagaCcauugugaaaacugau.................	1	1	Mm2
....ugagaucauugugaaaacuAau.................	1	1	Mm2
....ugagaucauugugaaaGcugau.................	35	1	Mm2
....ugagaucauugugaaaacugaG.................	4	1	Mm2
....Ngagaucauugugaaaacugau.................	17	1	Mm2
....ugagaucauugAgaaaacugau.................	2	1	Mm2
....ugagaucauugugaaaUcugau.................	48	1	Mm2
....ugagaucauugugCaaacugau.................	1	1	Mm2
....ugagaucCuugugaaaacugau.................	1	1	Mm2
....ugagaucauugugaaaacugGu.................	2	1	Mm2

Star Mature

```
ugauguuggcuguaaugaccuucagacgaaacugguuuucauaaugaccuuccagauuguugugcuauucugagaucauugugaaaacugauuccguugccccuaauc
......................................................................ugagaucaCugugaaaacugau................ 4 1 Mm2
......................................................................ugagaucauugugaGaacugau................ 1 1 Mm2
......................................................................ugagaucauuUugaaaacugau................ 16 1 Mm2
......................................................................ugagaucauugugaaaacugUu................ 6 1 Mm2
......................................................................ugagaucauugugaaaaUugau................ 146 1 Mm2
......................................................................ugagaucauugugaaaaGugau................ 118 1 Mm2
......................................................................ugagaucauGgugaaaacugau................ 3 1 Mm2
......................................................................ugagaucauugugaaaaAugau................ 247 1 Mm2
......................................................................ugagaucauugugaaaacugCu................ 1 1 Mm2
......................................................................ugUgaucauugugaaaacugau................ 4 1 Mm2
......................................................................ugGgaucauugugaaaacugau................ 8 1 Mm2
......................................................................ugaUaucauugugaaaacugau................ 1 1 Mm2
......................................................................ugagaNcauugugaaaacugau................ 4 1 Mm2
......................................................................ugagaucauugugaaaacGgau................ 2 1 Mm2
......................................................................ugagaucauuguCaaaacugau................ 2 1 Mm2
......................................................................ugagaucauugugaaCacugau................ 2 1 Mm2
......................................................................ugagaucauugugaaaacAgau................ 2 1 Mm2
......................................................................ugagaucauugugaaaacuUau................ 3 1 Mm2
......................................................................ugagauUauugugaaaacugau................ 6 1 Mm2
......................................................................ugagaucauugugaaaacugaC................ 8 1 Mm2
......................................................................ugagaucUuugugaaaacugau................ 8 1 Mm2
......................................................................ugagaucauugugaaaCcugau................ 49 1 Mm2
......................................................................ugagaucauuguAaaaacugau................ 3 1 Mm2
......................................................................ugagCucauugugaaaacugau................ 2 1 Mm2
......................................................................ugagGucauugugaaaacugau................ 4 1 Mm2
......................................................................ugagauGauugugaaaacugau................ 1 1 Mm2
......................................................................ugagauAauugugaaaacugau................ 2 1 Mm2
......................................................................Agagaucauugugaaaacugau................ 6 1 Mm2
......................................................................ugagaucauugugaaaacugaA................ 180 1 Mm2
......................................................................ugagaucauugugaaaacugau................ 27846 0 Mm2
......................................................................ugagaucauugugaaGacugau................ 2 1 Mm2
......................................................................ugagaucauugCgaaaacugau................ 7 1 Mm2
......................................................................ugagaucGuugugaaaacugau................ 28 1 Mm2
......................................................................ugagaucauugugUaaacugau................ 3 1 Mm2
......................................................................ugagaucauAgugaaaacugau................ 3 1 Mm2
......................................................................Cgagaucauugugaaaacugau................ 9 1 Mm2
......................................................................ugagaucauugugaaUacugau................ 1 1 Mm2
......................................................................ugagaGcauugugaaaacugau................ 1 1 Mm2
......................................................................ugagaucauugugGaaacugau................ 5 1 Mm2
......................................................................uUagaucauugugaaaacugau................ 10 1 Mm2
......................................................................ugagaucauugugaUaacugau................ 1 1 Mm2
......................................................................ugagaucauuAugaaaacugau................ 59 1 Mm2
......................................................................ugagaucauugugaaaacCgau................ 6 1 Mm2
......................................................................uAagaucauugugaaaacugau................ 13 1 Mm2
......................................................................ugagaucauuguCaaaacugauu............... 4 1 Mm2
......................................................................ugagaucauuAugaaaacugauu............... 60 1 Mm2
......................................................................ugagaucauugugGaaacugauu............... 3 1 Mm2
......................................................................ugagaucauugugaaaacGgauu............... 1 1 Mm2
......................................................................ugagaucauugugaaaacugauC............... 7 1 Mm2
......................................................................ugagaucaAugugaaaacugauu............... 5 1 Mm2
......................................................................Ggagaucauugugaaaacugauu............... 1 1 Mm2
......................................................................ugUgaucauugugaaaacugauu............... 4 1 Mm2
......................................................................Agagaucauugugaaaacugauu............... 6 1 Mm2
......................................................................ugagaucauuguAaaaacugauu............... 4 1 Mm2
......................................................................ugagaucauAgugaaaacugauu............... 2 1 Mm2
......................................................................ugaAaucauugugaaaacugauu............... 2 1 Mm2
......................................................................ugagaucauugugaUaacugauu............... 4 1 Mm2
......................................................................ugagaucauugugaaaacuUauu............... 7 1 Mm2
......................................................................ugagGucauugugaaaacugauu............... 3 1 Mm2
......................................................................uAagaucauugugaaaacugauu............... 22 1 Mm2
......................................................................ugagaucauugugaaaGcugauu............... 38 1 Mm2
......................................................................Cgagaucauugugaaaacugauu............... 10 1 Mm2
......................................................................ugagaucauuUugaaaacugauu............... 23 1 Mm2
......................................................................ugagauAauugugaaaacugauu............... 2 1 Mm2
......................................................................ugagauNauugugaaaacugauu............... 2 1 Mm2
......................................................................ugagaucauugugaaUacugauu............... 5 1 Mm2
......................................................................ugagaucauugugaaaaAugauu............... 282 1 Mm2
......................................................................ugaUaucauugugaaaacugauu............... 2 1 Mm2
......................................................................ugagaucauugugaaaacugaCu............... 2 1 Mm2
......................................................................uCagaucauugugaaaacugauu............... 4 1 Mm2
```

```
                                   Star                              Mature
ugauguuggcuguaaugaccuucagacgaaacugguuuucauaaugaccuuccagauuguugugcuauucugagaucauugugaaaacugauuccguugccccuaauc
......................................................................ugagCucauugugaaaacugauu...............    13      1   Mm2
......................................................................ugagaucauugugaaaCcugauu...............    69      1   Mm2
......................................................................ugagaucauugugaaaacugUuu...............    16      1   Mm2
......................................................................ugagaucauugugaaaaUugauu...............    173     1   Mm2
......................................................................ugagaucauugCgaaaacugauu...............    9       1   Mm2
......................................................................ugaCaucauugugaaaacugauu...............    1       1   Mm2
......................................................................ugagaNcauugugaaaacugauu...............    8       1   Mm2
......................................................................ugagaucauugugaaaacugCuu...............    1       1   Mm2
......................................................................ugagaucauugugaaaacugauu...............    34937   0   Mm2
......................................................................Ngagaucauugugaaaacugauu...............    18      1   Mm2
......................................................................ugagUucauugugaaaacugauu...............    8       1   Mm2
......................................................................ugagaucauugugaaGacugauu...............    5       1   Mm2
......................................................................ugagaucauGgugaaaacugauu...............    4       1   Mm2
......................................................................ugagaucauCgugaaaacugauu...............    3       1   Mm2
......................................................................ugagaucUuugugaaaacugauu...............    4       1   Mm2
......................................................................ugagaucauugugaaaacugauG...............    9       1   Mm2
......................................................................ugagaucaCugugaaaacugauu...............    3       1   Mm2
......................................................................ugagaucauuCugaaaacugauu...............    29      1   Mm2
......................................................................ugagaucauugugaaaUcugauu...............    70      1   Mm2
......................................................................ugagaucauugugaaaacAgauu...............    3       1   Mm2
......................................................................ugagaucauugugaaaacCgauu...............    6       1   Mm2
......................................................................ugagaucauugugaaaacugaAu...............    12      1   Mm2
......................................................................ugagaAcauugugaaaacugauu...............    1       1   Mm2
......................................................................ugagaucauugAgaaaacugauu...............    8       1   Mm2
......................................................................uUagaucauugugaaaacugauu...............    11      1   Mm2
......................................................................ugagaucauugugaaCacugauu...............    1       1   Mm2
......................................................................uNagaucauugugaaaacugauu...............    1       1   Mm2
......................................................................ugagaucauugugaaaacugGuu...............    6       1   Mm2
......................................................................ugagaucauugugaaaacugaGu...............    1       1   Mm2
......................................................................ugGgaucauugugaaaacugauu...............    14      1   Mm2
......................................................................ugagaucauugugaCaacugauu...............    1       1   Mm2
......................................................................ugagauUauugugaaaacugauu...............    11      1   Mm2
......................................................................ugagaucaGugugaaaacugauu...............    2       1   Mm2
......................................................................ugagaucauugugaGaacugauu...............    3       1   Mm2
......................................................................ugagaucGuugugaaaacugauu...............    90      1   Mm2
......................................................................ugagaCcauugugaaaacugauu...............    2       1   Mm2
......................................................................ugagaucauugugaaaacugauA...............    361     1   Mm2
......................................................................ugagaucauugugaaaaGugauu...............    140     1   Mm2
......................................................................ugagaucCuugugaaaacugauu...............    3       1   Mm2
......................................................................ugagaucauugugUaaacugauu...............    5       1   Mm2
......................................................................ugagaucauugugCaaacugauu...............    3       1   Mm2
......................................................................ugCgaucauugugaaaacugauu...............    5       1   Mm2
......................................................................ugagaucauugugaaaacugauuU..............    2922    1   Mm2
......................................................................ugagaucauugugaaaacugauuA..............    126     1   Mm2
......................................................................ugagaucauugugaaaaGugauuc..............    1       1   Mm2
......................................................................ugagaucauugugaaaacugauuG..............    4       1   Mm2
......................................................................ugagaucauugugaaaacugauuc..............    5       0   Mm2
......................................................................ugagaucauugugaaaacugauucU.............    1       1   Mm2
.......................................................................gGgaucauugugaaaacu...................    1       1   Mm2
.......................................................................gagaucauugugaaaacug..................    14      0   Mm2
.......................................................................gagaucauugugaaaacuga.................    2       0   Mm2
.......................................................................Aagaucauugugaaaacuga.................    1       1   Mm2
.......................................................................Uagaucauugugaaaacugau................    1       1   Mm2
.......................................................................gagaucauugugaaaacugau................    5       0   Mm2
.......................................................................Uagaucauugugaaaacugauu...............    1       1   Mm2
.......................................................................gagaucauugugaaaacugauu...............    4       0   Mm2
........................................................................Ugaucauugugaaaacug..................    1       1   Mm2
........................................................................Ugaucauugugaaaacugauu...............    1       1   Mm2

...............................cugguuuucauaaugaccuuccaga....................................................    2       0   Fm3
...................................uuuucauaaugaccuuAcag.....................................................    1       1   Fm3
...................................uuuucauaaugaccuuccaga....................................................    3       0   Fm3
..................................................................auucugagaucauugugaaaac....................    1       0   Fm3
...................................................................uucugagaucauugugaaa......................    10      0   Fm3
...................................................................uucugagaucauugugaaaac....................    2       0   Fm3
...................................................................uucugagaucauugugaGaacu...................    1       1   Fm3
...................................................................uucugagaucauugugUaaacu...................    2       1   Fm3
...................................................................uucugagaucauuAugaaaacu...................    1       1   Fm3
...................................................................Aucugagaucauugugaaaacu...................    1       1   Fm3
...................................................................uucugagaucauugugaaaacu...................    226     0   Fm3
```

Star Mature

ugauguuggcuguaaugaccuucagacgaaacugguuuucauaaugaccuuccagauuguugugcuauucugagaucauugugaaaacugauuccguugccccuaauc			
....uucugagaucauugugaaaGcu...................	1	1	Fm3
....uucugagaucauugugCaaacu...................	1	1	Fm3
....uAcugagaucauugugaaaacu...................	1	1	Fm3
....uucugagaucauugugaaaacuC..................	1	1	Fm3
....uucugagaucauugugaaaacuA..................	1	1	Fm3
....uucugagaucauugugaaaacug..................	3	0	Fm3
....ucugagaucauugugaaaacu...................	7	0	Fm3
....ucugagaucauugugaaaacug..................	4	0	Fm3
....cugagaucauugugaaaacug..................	2	0	Fm3
....cugagaucauugugaaaacugau................	2	0	Fm3
....cugagaucauugugaaaacugauu...............	2	0	Fm3
....ugagauUauugugaaaac....................	1	1	Fm3
....Agagaucauugugaaaac....................	1	1	Fm3
....ugaAaucauugugaaaac....................	1	1	Fm3
....ugagaucauugugaGaac....................	1	1	Fm3
....ugagaucauugugaaaCc....................	3	1	Fm3
....ugagaucauugugaaaGc....................	4	1	Fm3
....ugagCucauugugaaaac....................	1	1	Fm3
....ugUgaucauugugaaaac....................	1	1	Fm3
....ugagaNcauugugaaaac....................	1	1	Fm3
....ugagaucauuUugaaaac....................	2	1	Fm3
....ugagaucGuugugaaaac....................	1	1	Fm3
....uAagaucauugugaaaac....................	1	1	Fm3
....ugagaucauugugaaaUc....................	3	1	Fm3
....Cgagaucauugugaaaac....................	1	1	Fm3
....Ngagaucauugugaaaac....................	1	1	Fm3
....ugagaucauugugaaaac....................	1347	0	Fm3
....ugagaucauugugaaaaA....................	606	1	Fm3
....uUagaucauugugaaaac....................	1	1	Fm3
....ugagaucauuCugaaaac....................	3	1	Fm3
....ugagaucauugugaaaaU....................	214	1	Fm3
....ugagaucauugugaaaaG....................	564	1	Fm3
....ugaAaucauugugaaaacu...................	4	1	Fm3
....ugagaucauugugUaaacu...................	1	1	Fm3
....Agagaucauugugaaaacu...................	2	1	Fm3
....ugagauUauugugaaaacu...................	8	1	Fm3
....ugagaucauugugaaaaUu...................	168	1	Fm3
....ugagaucauCgugaaaacu...................	4	1	Fm3
....ugCgaucauugugaaaacu...................	4	1	Fm3
....ugUgaucauugugaaaacu...................	2	1	Fm3
....ugagaucaGugugaaaacu...................	3	1	Fm3
....ugagaucGuugugaaaacu...................	9	1	Fm3
....ugagaucauuguAaaaacu...................	2	1	Fm3
....ugagaucUuugugaaaacu...................	5	1	Fm3
....ugagaNcauugugaaaacu...................	1	1	Fm3
....ugagaucauugugaaaCcu...................	30	1	Fm3
....ugagaucauuUugaaaacu...................	27	1	Fm3
....ugagaucauugAgaaaacu...................	1	1	Fm3
....uUagaucauugugaaaacu...................	10	1	Fm3
....ugagauNauugugaaaacu...................	2	1	Fm3
....ugagaucauugugaaaacG...................	32	1	Fm3
....ugagaucauuguCaaaacu...................	1	1	Fm3
....uNagaucauugugaaaacu...................	2	1	Fm3
....Ngagaucauugugaaaacu...................	15	1	Fm3
....ugagaucauugugaGaacu...................	4	1	Fm3
....uAagaucauugugaaaacu...................	8	1	Fm3
....ugagCucauugugaaaacu...................	5	1	Fm3
....ugagaucauugCgaaaacu...................	12	1	Fm3
....Cgagaucauugugaaaacu...................	2	1	Fm3
....ugagaucauugugaaGacu...................	2	1	Fm3
....ugagauGauugugaaaacu...................	1	1	Fm3
....ugagaucauugugaaaacu...................	20479	0	Fm3
....ugagaAcauugugaaaacu...................	6	1	Fm3
....ugagauAauugugaaaacu...................	2	1	Fm3
....ugagaucauugugaaaaAu...................	288	1	Fm3
....uCagaucauugugaaaacu...................	2	1	Fm3
....ugagaucauugugaaaacA...................	30	1	Fm3
....ugagaucauugugaaUacu...................	1	1	Fm3
....ugagaucauugugaaaGcu...................	31	1	Fm3
....ugagaCcauugugaaaacu...................	1	1	Fm3

```
                                   Star                               Mature
ugauguuggcuguaaugaccuucagacgaaacugguuuucauaaugaccuuccagauuguugugcuauucugagaucauugugaaaacugauuccguugccccuaauc
......................................................................ugNgaucauugugaaaacu...................   2        1   Fm3
......................................................................ugagaucauugugGaaacu...................   2        1   Fm3
......................................................................ugagaucauuguUaaaacu...................   1        1   Fm3
......................................................................ugGgaucauugugaaaacu...................   6        1   Fm3
......................................................................ugagaucauuCugaaaacu...................   22       1   Fm3
......................................................................ugagaucauugugaaaUcu...................   40       1   Fm3
......................................................................ugagaucauugugaaaaGu...................   108      1   Fm3
......................................................................ugagUucauugugaaaacu...................   5        1   Fm3
......................................................................ugaUaucauugugaaaacu...................   3        1   Fm3
......................................................................ugagGucauugugaaaacu...................   2        1   Fm3
......................................................................ugaCaucauugugaaaacu...................   1        1   Fm3
......................................................................ugagaucauugugaaaaUug..................   673      1   Fm3
......................................................................ugagaucauugugaaaacGg..................   4        1   Fm3
......................................................................ugagaucauGgugaaaacug..................   8        1   Fm3
......................................................................ugagaNcauugugaaaacug..................   27       1   Fm3
......................................................................ugUgaucauugugaaaacug..................   24       1   Fm3
......................................................................ugagaucauugugaaaacuU..................   124      1   Fm3
......................................................................ugagauAauugugaaaacug..................   8        1   Fm3
......................................................................Cgagaucauugugaaaacug..................   20       1   Fm3
......................................................................ugagaucauugugaaaacuC..................   9        1   Fm3
......................................................................ugagaucauugugaaaCcug..................   221      1   Fm3
......................................................................ugagaucauugugaCaacug..................   1        1   Fm3
......................................................................ugagaucauugugaaaaAug..................   1192     1   Fm3
......................................................................ugagaucauugugaaaacAg..................   4        1   Fm3
......................................................................ugagaucGuugugaaaacug..................   22       1   Fm3
......................................................................ugGgaucauugugaaaacug..................   56       1   Fm3
......................................................................ugagauGauugugaaaacug..................   7        1   Fm3
......................................................................ugagauUauugugaaaacug..................   58       1   Fm3
......................................................................ugagaucauugugGaaacug..................   20       1   Fm3
......................................................................ugagaucauugugaUaacug..................   19       1   Fm3
......................................................................ugagaAcauugugaaaacug..................   9        1   Fm3
......................................................................ugCgaucauugugaaaacug..................   15       1   Fm3
......................................................................ugagaucauugugaaaGcug..................   181      1   Fm3
......................................................................ugagCucauugugaaaacug..................   25       1   Fm3
......................................................................ugagaucauAgugaaaacug..................   8        1   Fm3
......................................................................ugagaucaGugugaaaacug..................   9        1   Fm3
......................................................................ugagaucUuugugaaaacug..................   23       1   Fm3
......................................................................ugagaucauugugaaUacug..................   5        1   Fm3
......................................................................ugagaucauuUugaaaacug..................   92       1   Fm3
......................................................................ugagaucauugugaaaacCg..................   27       1   Fm3
......................................................................ugagaucauugugaaaaGug..................   630      1   Fm3
......................................................................ugagaucauugugaGaacug..................   23       1   Fm3
......................................................................ugagGucauugugaaaacug..................   43       1   Fm3
......................................................................ugagaucauugugaaaUcug..................   270      1   Fm3
......................................................................ugagaucauuCugaaaacug..................   146      1   Fm3
......................................................................ugNgaucauugugaaaacug..................   1        1   Fm3
......................................................................ugagaucaAugugaaaacug..................   6        1   Fm3
......................................................................ugaUaucauugugaaaacug..................   13       1   Fm3
......................................................................uCagaucauugugaaaacug..................   20       1   Fm3
......................................................................ugagaucauugugaaCacug..................   3        1   Fm3
......................................................................ugagauNauugugaaaacug..................   4        1   Fm3
......................................................................ugagaucauCgugaaaacug..................   11       1   Fm3
......................................................................uNagaucauugugaaaacug..................   4        1   Fm3
......................................................................Ngagaucauugugaaaacug..................   66       1   Fm3
......................................................................ugagaucauuguUaaaacug..................   20       1   Fm3
......................................................................ugagaucauugugUaaacug..................   33       1   Fm3
......................................................................ugagaGcauugugaaaacug..................   15       1   Fm3
......................................................................ugagaucauugGgaaaacug..................   5        1   Fm3
......................................................................ugagaucauuguCaaaacug..................   6        1   Fm3
......................................................................ugagaucauuguAaaaacug..................   18       1   Fm3
......................................................................ugagaucauugCgaaaacug..................   26       1   Fm3
......................................................................uUagaucauugugaaaacug..................   42       1   Fm3
......................................................................ugagaucauugugaaGacug..................   19       1   Fm3
......................................................................ugagaucauugAgaaaacug..................   12       1   Fm3
......................................................................ugagaucauugugCaaacug..................   7        1   Fm3
......................................................................ugagaucaCugugaaaacug..................   13       1   Fm3
......................................................................ugagaucauugugaaaacug..................   142469   0   Fm3
......................................................................ugagaucauugugaaaacuA..................   550      1   Fm3
......................................................................uAagaucauugugaaaacug..................   43       1   Fm3
......................................................................ugaAaucauugugaaaacug..................   36       1   Fm3
```

Star Mature

```
ugauguuggcuguaaugaccuucagacgaaacugguuuucauaaugaccuuccagauuguugugcuauucugagaucauugugaaaacugauuccguugccccuaauc
......................................................................ugagaCcauugugaaaacug..................    23     1    Fm3
......................................................................Ggagaucauugugaaaacug..................    4      1    Fm3
......................................................................ugagaucauuAugaaaacug..................    276    1    Fm3
......................................................................Agagaucauugugaaaacug..................    13     1    Fm3
......................................................................ugagUucauugugaaaacug..................    14     1    Fm3
......................................................................ugaCaucauugugaaaacug..................    10     1    Fm3
......................................................................ugagauUauugugaaaacuga.................    32     1    Fm3
......................................................................ugagauAauugugaaaacuga.................    2      1    Fm3
......................................................................ugagaucauugugaaaacuAa.................    11     1    Fm3
......................................................................ugagaucauugugaaaacCga.................    13     1    Fm3
......................................................................ugagauGauugugaaaacuga.................    7      1    Fm3
......................................................................ugagaucauugugaGaacuga.................    17     1    Fm3
......................................................................ugagaucauugugCaaacuga.................    5      1    Fm3
......................................................................ugagCucauugugaaaacuga.................    21     1    Fm3
......................................................................ugagaucauugugaaaUcuga.................    177    1    Fm3
......................................................................uCagaucauugugaaaacuga.................    16     1    Fm3
......................................................................ugagaAcauugugaaaacuga.................    5      1    Fm3
......................................................................ugagaucaCugugaaaacuga.................    7      1    Fm3
......................................................................uAagaucauugugaaaacuga.................    58     1    Fm3
......................................................................Agagaucauugugaaaacuga.................    5      1    Fm3
......................................................................ugaCaucauugugaaaacuga.................    5      1    Fm3
......................................................................ugagaucauugugaaaacuga.................    99043  0    Fm3
......................................................................ugagaucauuUugaaaacuga.................    71     1    Fm3
......................................................................ugagaucauugugaaaacugG.................    10     1    Fm3
......................................................................ugagUucauugugaaaacuga.................    15     1    Fm3
......................................................................ugagaucauuCugaaaacuga.................    101    1    Fm3
......................................................................ugCgaucauugugaaaacuga.................    14     1    Fm3
......................................................................ugagaucauugugGaaacuga.................    16     1    Fm3
......................................................................ugagaucauugugaaaaGuga.................    459    1    Fm3
......................................................................ugagaucauGgugaaaacuga.................    9      1    Fm3
......................................................................ugagaucauugugaaaacuUa.................    15     1    Fm3
......................................................................ugagaGcauugugaaaacuga.................    5      1    Fm3
......................................................................ugagaucaGugugaaaacuga.................    7      1    Fm3
......................................................................ugagaucauuAugaaaacuga.................    192    1    Fm3
......................................................................ugagaucCuugugaaaacuga.................    1      1    Fm3
......................................................................ugGgaucauugugaaaacuga.................    45     1    Fm3
......................................................................ugagGucauugugaaaacuga.................    21     1    Fm3
......................................................................ugagauNauugugaaaacuga.................    10     1    Fm3
......................................................................ugagaucauugugUaaacuga.................    16     1    Fm3
......................................................................Ngagaucauugugaaaacuga.................    40     1    Fm3
......................................................................ugagaucauugugaaaacugC.................    3      1    Fm3
......................................................................ugagaucauugugaaaaAuga.................    798    1    Fm3
......................................................................ugagaucauAgugaaaacuga.................    6      1    Fm3
......................................................................ugaAaucauugugaaaacuga.................    16     1    Fm3
......................................................................ugaUaucauugugaaaacuga.................    14     1    Fm3
......................................................................ugagaucauugCgaaaacuga.................    13     1    Fm3
......................................................................ugagaNcauugugaaaacuga.................    14     1    Fm3
......................................................................ugagaucauugugaaaaUuga.................    500    1    Fm3
......................................................................ugagaucaAugugaaaacuga.................    1      1    Fm3
......................................................................ugUgaucauugugaaaacuga.................    14     1    Fm3
......................................................................ugagaucauuguUaaaacuga.................    15     1    Fm3
......................................................................ugagaucauugugaUaacuga.................    6      1    Fm3
......................................................................ugagaucUuugugaaaacuga.................    12     1    Fm3
......................................................................ugagaucauugugaaaCcuga.................    166    1    Fm3
......................................................................ugagaucauugugaaaacugU.................    1359   1    Fm3
......................................................................ugagaucauugugaaGacuga.................    13     1    Fm3
......................................................................ugagaucauugugaaaacAga.................    4      1    Fm3
......................................................................ugagaucauugugaaaacGga.................    5      1    Fm3
......................................................................ugagaCcauugugaaaacuga.................    8      1    Fm3
......................................................................ugagaucGuugugaaaacuga.................    26     1    Fm3
......................................................................ugagaucauugAgaaaacuga.................    13     1    Fm3
......................................................................Cgagaucauugugaaaacuga.................    14     1    Fm3
......................................................................Ggagaucauugugaaaacuga.................    2      1    Fm3
......................................................................ugagaucauugugaCaacuga.................    4      1    Fm3
......................................................................ugagaucauugugaaaacuCa.................    5      1    Fm3
......................................................................ugagaucauuguCaaaacuga.................    16     1    Fm3
......................................................................ugagaucauCgugaaaacuga.................    10     1    Fm3
......................................................................ugagaucauugGgaaaacuga.................    2      1    Fm3
......................................................................ugagaucauugugaaaGcuga.................    116    1    Fm3
......................................................................uUagaucauugugaaaacuga.................    34     1    Fm3
```

```
                                           Star                                     Mature
ugauguuggcuguaaugaccuucagacgaaacugguuuucauaaugaccuuccagauuguugugcuauucugagaucauugugaaaacugauuccguugccccuaauc
......................................................................ugagaucauugugaaUacuga.................   8       1   Fm3
......................................................................ugagaucauuguAaaaacuga.................   8       1   Fm3
......................................................................ugagaucauugugaaCacuga.................   1       1   Fm3
......................................................................ugagaucauAgugaaaacugau................   3       1   Fm3
......................................................................ugagaucauuUugaaaacugau................   43      1   Fm3
......................................................................ugagaucauuCugaaaacugau................   69      1   Fm3
......................................................................ugagaucauugugaaaacugaC................   6       1   Fm3
......................................................................ugagaucauugugaaaacugaA................   714     1   Fm3
......................................................................ugagaucauCgugaaaacugau................   5       1   Fm3
......................................................................ugagaucauugugaGaacugau................   13      1   Fm3
......................................................................ugagaucCuugugaaaacugau................   1       1   Fm3
......................................................................ugagaucauGgugaaaacugau................   4       1   Fm3
......................................................................ugagaucauugugaaaUcugau................   125     1   Fm3
......................................................................ugagaucauugugaaaacuAau................   6       1   Fm3
......................................................................ugagaucauugugaaaacugCu................   1       1   Fm3
......................................................................ugagaucaGugugaaaacugau................   3       1   Fm3
......................................................................ugagauGauugugaaaacugau................   3       1   Fm3
......................................................................ugagaucauugugaaaGcugau................   75      1   Fm3
......................................................................ugaAaucauugugaaaacugau................   10      1   Fm3
......................................................................ugaCaucauugugaaaacugau................   6       1   Fm3
......................................................................ugagCucauugugaaaacugau................   7       1   Fm3
......................................................................Ggagaucauugugaaaacugau................   1       1   Fm3
......................................................................ugagauUauugugaaaacugau................   29      1   Fm3
......................................................................ugagaucauugugaaUacugau................   4       1   Fm3
......................................................................ugagaucauugugaaCacugau................   2       1   Fm3
......................................................................ugagaucauugugaaaacugUu................   15      1   Fm3
......................................................................ugagaucaCugugaaaacugau................   7       1   Fm3
......................................................................ugagaucauugugCaaacugau................   5       1   Fm3
......................................................................ugagaNcauugugaaaacugau................   10      1   Fm3
......................................................................ugGgaucauugugaaaacugau................   22      1   Fm3
......................................................................ugagaucauugugaCaacugau................   1       1   Fm3
......................................................................ugagaucauugugaaaacugGu................   5       1   Fm3
......................................................................ugaUaucauugugaaaacugau................   7       1   Fm3
......................................................................ugagaucauugugaaaCcugau................   85      1   Fm3
......................................................................ugagaucauugugaaGacugau................   5       1   Fm3
......................................................................ugagaucauugugaaaacuUau................   11      1   Fm3
......................................................................uNagaucauugugaaaacugau................   3       1   Fm3
......................................................................ugagaucauugugaaaacugau................   58032   0   Fm3
......................................................................ugagaucauugugaaaaAugau................   510     1   Fm3
......................................................................ugagaAcauugugaaaacugau................   4       1   Fm3
......................................................................ugagauNauugugaaaacugau................   4       1   Fm3
......................................................................ugagaucauugugaaaaGugau................   244     1   Fm3
......................................................................ugagaucaAugugaaaacugau................   5       1   Fm3
......................................................................ugagaucauugugaaaacAgau................   6       1   Fm3
......................................................................ugagaucUuugugaaaacugau................   9       1   Fm3
......................................................................ugagaucauuguAaaaacugau................   7       1   Fm3
......................................................................ugagauAauugugaaaacugau................   1       1   Fm3
......................................................................ugagaucauugugaaaacGgau................   2       1   Fm3
......................................................................Cgagaucauugugaaaacugau................   6       1   Fm3
......................................................................Agagaucauugugaaaacugau................   3       1   Fm3
......................................................................ugagaucauugCgaaaacugau................   14      1   Fm3
......................................................................ugagaucauugGgaaaacugau................   7       1   Fm3
......................................................................ugCgaucauugugaaaacugau................   4       1   Fm3
......................................................................ugagaucauugugGaaacugau................   10      1   Fm3
......................................................................ugagGucauugugaaaacugau................   8       1   Fm3
......................................................................Ngagaucauugugaaaacugau................   31      1   Fm3
......................................................................ugagaCcauugugaaaacugau................   3       1   Fm3
......................................................................ugagUucauugugaaaacugau................   11      1   Fm3
......................................................................ugagaucauugugaaaaUugau................   280     1   Fm3
......................................................................uAagaucauugugaaaacugau................   25      1   Fm3
......................................................................ugagaucauuguUaaaacugau................   9       1   Fm3
......................................................................ugUgaucauugugaaaacugau................   12      1   Fm3
......................................................................uCagaucauugugaaaacugau................   3       1   Fm3
......................................................................ugagaucauuAugaaaacugau................   117     1   Fm3
......................................................................ugagaucauugugaaaacuCau................   3       1   Fm3
......................................................................ugagaucauugugaaaacCgau................   5       1   Fm3
......................................................................ugagaucGuugugaaaacugau................   18      1   Fm3
......................................................................ugagaucauugAgaaaacugau................   7       1   Fm3
......................................................................ugagaucauuguCaaaacugau................   7       1   Fm3
......................................................................ugagaucauugugUaaacugau................   10      1   Fm3
```

```
                                      Star                                Mature
ugauguuggcuguaaugaccuucagacgaaacugguuuucauaaugaccuuccagauuguugugcuauucugagaucauugugaaaacugauuccguugccccuaauc
.....................................................................ugagaucauugugaaaacugaG.................   1       1    Fm3
.....................................................................uUagaucauugugaaaacugau.................   21      1    Fm3
.....................................................................ugagaucauugugaaaacCgauu................   10      1    Fm3
.....................................................................ugagaucauugugaaaaGugauu................   262     1    Fm3
.....................................................................ugagaucauugugaaaacugGuu................   7       1    Fm3
.....................................................................ugagaucauugugaUaacugauu................   6       1    Fm3
.....................................................................ugagaucauAgugaaaacugauu................   4       1    Fm3
.....................................................................Agagaucauugugaaaacugauu................   6       1    Fm3
.....................................................................ugagaucaGugugaaaacugauu................   4       1    Fm3
.....................................................................ugagaucauugugGaaacugauu................   10      1    Fm3
.....................................................................ugagaCcauugugaaaacugauu................   5       1    Fm3
.....................................................................ugagaucUuugugaaaacugauu................   8       1    Fm3
.....................................................................ugagaucauugugaaaacugauu................   58028   0    Fm3
.....................................................................ugagaucauugugaaCacugauu................   1       1    Fm3
.....................................................................ugagaAcauugugaaaacugauu................   8       1    Fm3
.....................................................................ugagaucauuCugaaaacugauu................   55      1    Fm3
.....................................................................ugagaucauugugaaaUcugauu................   108     1    Fm3
.....................................................................ugagaucauCgugaaaacugauu................   8       1    Fm3
.....................................................................ugCgaucauugugaaaacugauu................   7       1    Fm3
.....................................................................ugaCaucauugugaaaacugauu................   5       1    Fm3
.....................................................................ugagaucauugugaaaacuAauu................   4       1    Fm3
.....................................................................ugagaucGuugugaaaacugauu................   14      1    Fm3
.....................................................................ugagaucauugugaaaCcugauu................   86      1    Fm3
.....................................................................ugagauNauugugaaaacugauu................   7       1    Fm3
.....................................................................ugagaucauugugaaaacugauG................   3       1    Fm3
.....................................................................ugaAaucauugugaaaacugauu................   7       1    Fm3
.....................................................................ugaUaucauugugaaaacugauu................   5       1    Fm3
.....................................................................ugagaucauugugaaaaAugauu................   501     1    Fm3
.....................................................................Ggagaucauugugaaaacugauu................   2       1    Fm3
.....................................................................ugagaucauugCgaaaacugauu................   11      1    Fm3
.....................................................................ugagaucauugugUaaacugauu................   12      1    Fm3
.....................................................................ugagUucauugugaaaacugauu................   8       1    Fm3
.....................................................................ugagauGauugugaaaacugauu................   5       1    Fm3
.....................................................................ugagaucauugugaaaacugauA................   221     1    Fm3
.....................................................................ugagGucauugugaaaacugauu................   14      1    Fm3
.....................................................................ugagaucauugugaaaacuCauu................   4       1    Fm3
.....................................................................ugagaucauugugaaaGcugauu................   74      1    Fm3
.....................................................................ugagaucauugugaaaacAgauu................   2       1    Fm3
.....................................................................uCagaucauugugaaaacugauu................   7       1    Fm3
.....................................................................ugagaucauugugaaaacugaGu................   2       1    Fm3
.....................................................................ugagaucauugugaaaacugaAu................   27      1    Fm3
.....................................................................ugagaucauugugaaGacugauu................   13      1    Fm3
.....................................................................ugagaGcauugugaaaacugauu................   3       1    Fm3
.....................................................................ugagaucaAugugaaaacugauu................   2       1    Fm3
.....................................................................ugagaucauugugaaaacuUauu................   9       1    Fm3
.....................................................................ugagauUauugugaaaacugauu................   17      1    Fm3
.....................................................................ugagaucauugugaGaacugauu................   16      1    Fm3
.....................................................................ugUgaucauugugaaaacugauu................   12      1    Fm3
.....................................................................ugagaucauugugaaaacugCuu................   1       1    Fm3
.....................................................................ugagaucauugugaaaacugauC................   3       1    Fm3
.....................................................................uUagaucauugugaaaacugauu................   16      1    Fm3
.....................................................................Ngagaucauugugaaaacugauu................   26      1    Fm3
.....................................................................ugagaucauGgugaaaacugauu................   4       1    Fm3
.....................................................................ugagaucauuguAaaaacugauu................   11      1    Fm3
.....................................................................ugagaucauugugCaaacugauu................   4       1    Fm3
.....................................................................ugagaucauuguUaaaacugauu................   8       1    Fm3
.....................................................................ugagaucauugugaaaacugaCu................   2       1    Fm3
.....................................................................ugagaNcauugugaaaacugauu................   7       1    Fm3
.....................................................................Cgagaucauugugaaaacugauu................   15      1    Fm3
.....................................................................ugNgaucauugugaaaacugauu................   1       1    Fm3
.....................................................................ugGgaucauugugaaaacugauu................   19      1    Fm3
.....................................................................ugagauAauugugaaaacugauu................   3       1    Fm3
.....................................................................ugagCucauugugaaaacugauu................   13      1    Fm3
.....................................................................uAagaucauugugaaaacugauu................   25      1    Fm3
.....................................................................ugagaucauugugaaaaUugauu................   284     1    Fm3
.....................................................................ugagaucauuguCaaaacugauu................   4       1    Fm3
.....................................................................ugagaucauugugaaaacugUuu................   4       1    Fm3
.....................................................................ugagaucaCugugaaaacugauu................   2       1    Fm3
.....................................................................ugagaucauugAgaaaacugauu................   7       1    Fm3
.....................................................................ugagaucauuUugaaaacugauu................   55      1    Fm3
```

Star **Mature**

```
ugauguuggcuguaaugaccuucagacgaaacugguuuucauaaugaccuuccagauuguugugcuauucugagaucauugugaaaacugauuccguugccccuaauc
......................................................................ugagaucauugugaaUacugauu...............   7     1   Fm3
......................................................................ugagaucauugGgaaaacugauu...............   1     1   Fm3
......................................................................ugagaucauuAugaaaacugauu...............   123   1   Fm3
......................................................................ugagaucauugugaaaacugauuU..............   4874  1   Fm3
......................................................................ugagaucauugugaaaacugauuA..............   138   1   Fm3
......................................................................ugagaucauugugaaaacugauuG..............   2     1   Fm3
......................................................................ugagaucauugugaaaacugauuc..............   6     0   Fm3
.......................................................................Uagaucauugugaaaacu...................   1     1   Fm3
.......................................................................gagaucauugugaaaacug..................   95    0   Fm3
.......................................................................Uagaucauugugaaaacug..................   2     1   Fm3
.......................................................................gagaucauuAugaaaacug..................   1     1   Fm3
.......................................................................gagaucauuUugaaaacug..................   2     1   Fm3
.......................................................................gagaucauugugaaaacuga.................   8     0   Fm3
.......................................................................gagaucauugugaaaacugU.................   1     1   Fm3
.......................................................................gagaucauugugaaaacugau................   6     0   Fm3
.......................................................................Uagaucauugugaaaacugau................   1     1   Fm3
.......................................................................gagaucauugugaaaacugaA................   1     1   Fm3
.......................................................................gagaucauugugaaaacugUuu...............   1     1   Fm3
.......................................................................gagaucauugugaaaacugauu...............   14    0   Fm3
.......................................................................Uagaucauugugaaaacugauu...............   2     1   Fm3
.......................................................................gagaucauugugaaaacugauuU..............   1     1   Fm3
........................................................................Ugaucauugugaaaacug..................   6     1   Fm3
........................................................................Ugaucauugugaaaacugau................   1     1   Fm3
........................................................................Ugaucauugugaaaacugauu...............   4     1   Fm3

...............................cugguuuucauaaugaccuucc.......................................................   5     0   Fm1
...............................cugguuuucauaaugaccuucG.......................................................   1     1   Fm1
...............................cugguuuucauaaugaccuucca......................................................   3     0   Fm1
...............................cugguuuucauaaugaccuuccG......................................................   1     1   Fm1
...............................cugguuuucauaaugacUuuccag.....................................................   2     1   Fm1
...............................cugguuuucaGaaugaccuuccag.....................................................   1     1   Fm1
...............................cugguuuucauaaugaccuuccag.....................................................   35    0   Fm1
...............................cugguuuucauaaugacAuuccaga....................................................   1     1   Fm1
...............................cugguNuucauaaugaccuuccaga....................................................   1     1   Fm1
...............................cugguuuucauaaugaccuuccagU....................................................   4     1   Fm1
...............................cugguuuucauaaugaccuuccaga....................................................   119   0   Fm1
...............................cugguuuucauaaugacUuuccaga....................................................   1     1   Fm1
...............................cugguuuucaCaaugaccuuccaga....................................................   2     1   Fm1
.................................gguuuucauaaugaccuuccagU....................................................   4     1   Fm1
...................................uuuucauaaugaccuuccaga....................................................   5     0   Fm1
...................................................................uucugagaucauugugaaa......................   6     0   Fm1
...................................................................uucugagaucauugugaaaa.....................   3     0   Fm1
...................................................................uucugagaucauugugaaaaU....................   5     1   Fm1
...................................................................uucugagaucauugugaaaac....................   10    0   Fm1
...................................................................uucugagaucauugugUaaacu...................   1     1   Fm1
...................................................................uucugagaucauugugaaaaUu...................   1     1   Fm1
...................................................................uucugagaucauugugCaaacu...................   2     1   Fm1
...................................................................uucuAagaucauugugaaaacu...................   2     1   Fm1
...................................................................uucugagaucauugGgaaaacu...................   1     1   Fm1
...................................................................uucugagaucUuugugaaaacu...................   2     1   Fm1
...................................................................uuGugagaucauugugaaaacu...................   1     1   Fm1
...................................................................uucugagaucauugugaUaacu...................   1     1   Fm1
...................................................................Cucugagaucauugugaaaacu...................   2     1   Fm1
...................................................................uucugagaucGuugugaaaacu...................   6     1   Fm1
...................................................................uucugagaucauugugaaaacG...................   3     1   Fm1
...................................................................uucugagaucauugugaGaacu...................   2     1   Fm1
...................................................................uucugagaucCuugugaaaacu...................   1     1   Fm1
...................................................................uuAugagaucauugugaaaacu...................   1     1   Fm1
...................................................................uucugGgaucauugugaaaacu...................   1     1   Fm1
...................................................................uucugagaucauugugaaaacu...................   636   0   Fm1
...................................................................uAcugagaucauugugaaaacu...................   1     1   Fm1
...................................................................uucugagaucauugugaaaacuU..................   6     1   Fm1
...................................................................uucugagaucauugugaUaacug..................   1     1   Fm1
...................................................................uucugagaucauugugaaaacuA..................   8     1   Fm1
...................................................................uucugagaucauugugaaaacug..................   32    0   Fm1
...................................................................uucugagaucauugugGaaacug..................   1     1   Fm1
....................................................................ucugagaucauugugaaaacug..................   7     0   Fm1
.....................................................................cugagaucauugugaaaacGg..................   1     1   Fm1
.....................................................................cugagaucauugugaaaacug..................   5     0   Fm1
.....................................................................cugagaucauugugaaaacugau................   6     0   Fm1
```

Star Mature

```
ugauguuggcuguaaugaccuucagacgaaacugguuuucauaaugaccuuccagauuguugugcuauucugagaucauugugaaaacugauuccguugccccuaauc
......................................................................ugagaucauugugaaaGc....................    1       1    Fm1
......................................................................ugagaucauugugaaaaU....................    24      1    Fm1
......................................................................ugagaucauuCugaaaac....................    1       1    Fm1
......................................................................ugagaucauugugaaaaA....................    133     1    Fm1
......................................................................ugagaucauugugaaaaG....................    106     1    Fm1
......................................................................ugagaucGuugugaaaac....................    3       1    Fm1
......................................................................ugagaucauugugaaaac....................    186     0    Fm1
......................................................................ugagauNauugugaaaac....................    1       1    Fm1
......................................................................ugagaucauugugaaaGcu...................    10      1    Fm1
......................................................................uUagaucauugugaaaacu...................    1       1    Fm1
......................................................................uAagaucauugugaaaacu...................    7       1    Fm1
......................................................................ugagaucauugugaaaaGu...................    52      1    Fm1
......................................................................ugagaucauuguAaaaacu...................    3       1    Fm1
......................................................................ugagCucauugugaaaacu...................    3       1    Fm1
......................................................................ugaCaucauugugaaaacu...................    2       1    Fm1
......................................................................ugagaucauugugaaaacC...................    3       1    Fm1
......................................................................ugagaucauugugaaaCcu...................    14      1    Fm1
......................................................................ugGgaucauugugaaaacu...................    3       1    Fm1
......................................................................ugCgaucauugugaaaacu...................    2       1    Fm1
......................................................................ugUgaucauugugaaaacu...................    5       1    Fm1
......................................................................ugagaucCuugugaaaacu...................    2       1    Fm1
......................................................................ugagaucauugugaaGacu...................    3       1    Fm1
......................................................................ugagaucauugugGaaacu...................    1       1    Fm1
......................................................................ugagaucauuguUaaaacu...................    2       1    Fm1
......................................................................ugagaCcauugugaaaacu...................    1       1    Fm1
......................................................................ugagaucauCgugaaaacu...................    1       1    Fm1
......................................................................ugagaucauugugaaaacA...................    24      1    Fm1
......................................................................ugagGucauugugaaaacu...................    4       1    Fm1
......................................................................ugagaucauugugaaaaUu...................    65      1    Fm1
......................................................................ugagaAcauugugaaaacu...................    5       1    Fm1
......................................................................ugagaucauugugaUaacu...................    1       1    Fm1
......................................................................ugagUucauugugaaaacu...................    1       1    Fm1
......................................................................ugagaucauugugaaaUcu...................    25      1    Fm1
......................................................................ugagaucauugCgaaaacu...................    1       1    Fm1
......................................................................ugagaucUuugugaaaacu...................    2       1    Fm1
......................................................................ugagaucauuCugaaaacu...................    10      1    Fm1
......................................................................ugagaucauugugaaaacG...................    117     1    Fm1
......................................................................Ngagaucauugugaaaacu...................    4       1    Fm1
......................................................................ugagaNcauugugaaaacu...................    3       1    Fm1
......................................................................ugagaucauuUugaaaacu...................    6       1    Fm1
......................................................................ugagaucaCugugaaaacu...................    1       1    Fm1
......................................................................ugagauAauugugaaaacu...................    2       1    Fm1
......................................................................ugagaucauugugaGaacu...................    11      1    Fm1
......................................................................ugagaucauugugaaaacu...................    10860   0    Fm1
......................................................................ugagauNauugugaaaacu...................    2       1    Fm1
......................................................................ugagaucGuugugaaaacu...................    45      1    Fm1
......................................................................ugagaucauAgugaaaacu...................    1       1    Fm1
......................................................................ugagauUauugugaaaacu...................    6       1    Fm1
......................................................................ugagaucauugAgaaaacu...................    3       1    Fm1
......................................................................ugagaucauugugaaaaAu...................    114     1    Fm1
......................................................................Cgagaucauugugaaaacu...................    3       1    Fm1
......................................................................ugagaucaAugugaaaacu...................    2       1    Fm1
......................................................................ugaUaucauugugaaaacug..................    13      1    Fm1
......................................................................ugaAaucauugugaaaacug..................    30      1    Fm1
......................................................................ugagaucauuguAaaaacug..................    27      1    Fm1
......................................................................ugagaucauuguCaaaacug..................    9       1    Fm1
......................................................................ugagaucaAugugaaaacug..................    6       1    Fm1
......................................................................ugagaucauugugaaGacug..................    40      1    Fm1
......................................................................ugagUucauugugaaaacug..................    25      1    Fm1
......................................................................ugagaucauugugaaaacug..................    195850  0    Fm1
......................................................................ugagauAauugugaaaacug..................    10      1    Fm1
......................................................................ugagaucauuCugaaaacug..................    181     1    Fm1
......................................................................ugagaucauugugaaaaAug..................    1638    1    Fm1
......................................................................ugagaGcauugugaaaacug..................    8       1    Fm1
......................................................................ugUgaucauugugaaaacug..................    37      1    Fm1
......................................................................ugagaucauugugaCaacug..................    5       1    Fm1
......................................................................ugagaucauCgugaaaacug..................    29      1    Fm1
......................................................................ugagaCcauugugaaaacug..................    8       1    Fm1
......................................................................ugagaucauuAugaaaacug..................    418     1    Fm1
......................................................................Agagaucauugugaaaacug..................    24      1    Fm1
```

Star **Mature**

```
ugauguuggcuguaaugaccuucagacgaaacugguuuucauaaugaccuuccagauuguugugcuauucugagaucauugugaaaacugauuccguugccccuaauc
......................................................................ugGgaucauugugaaaacug..................  69     1  Fm1
......................................................................ugagaucauugugaaaUcug..................  376    1  Fm1
......................................................................ugagaNcauugugaaaacug..................  30     1  Fm1
......................................................................ugagaucauugugaaaaUug..................  979    1  Fm1
......................................................................ugagaucaCugugaaaacug..................  13     1  Fm1
......................................................................ugagaucGuugugaaaacug..................  44     1  Fm1
......................................................................ugagaucCuugugaaaacug..................  3      1  Fm1
......................................................................ugagaucauugugaaaacGg..................  1343   1  Fm1
......................................................................ugagaucauugugaaaCcug..................  375    1  Fm1
......................................................................ugagaucauugugaaaacAg..................  12     1  Fm1
......................................................................ugagaucauugugaaaacuC..................  4      1  Fm1
......................................................................ugagCucauugugaaaacug..................  22     1  Fm1
......................................................................ugagauGauugugaaaacug..................  4      1  Fm1
......................................................................ugaCaucauugugaaaacug..................  10     1  Fm1
......................................................................Cgagaucauugugaaaacug..................  64     1  Fm1
......................................................................ugagaucauugCgaaaacug..................  30     1  Fm1
......................................................................ugagaucauugugaUaacug..................  12     1  Fm1
......................................................................ugagaucauugugaaaacCg..................  31     1  Fm1
......................................................................Ggagaucauugugaaaacug..................  8      1  Fm1
......................................................................ugagaAcauugugaaaacug..................  17     1  Fm1
......................................................................uCagaucauugugaaaacug..................  16     1  Fm1
......................................................................ugagGucauugugaaaacug..................  27     1  Fm1
......................................................................ugagaucaGugugaaaacug..................  13     1  Fm1
......................................................................uAagaucauugugaaaacug..................  62     1  Fm1
......................................................................ugagauNauugugaaaacug..................  18     1  Fm1
......................................................................ugagaucauugugaaaaGug..................  868    1  Fm1
......................................................................ugagaucauGgugaaaacug..................  10     1  Fm1
......................................................................Ngagaucauugugaaaacug..................  94     1  Fm1
......................................................................uNagaucauugugaaaacug..................  1      1  Fm1
......................................................................ugagaucauugugaGaacug..................  15     1  Fm1
......................................................................ugagaucauugugaaaacuA..................  399    1  Fm1
......................................................................ugagaucauugugGaaacug..................  36     1  Fm1
......................................................................ugagaucauugugaaaaNug..................  1      1  Fm1
......................................................................ugagaucauugGgaaaacug..................  6      1  Fm1
......................................................................ugagaucUuugugaaaacug..................  40     1  Fm1
......................................................................ugagaucauAgugaaaacug..................  12     1  Fm1
......................................................................ugagaucauugugaaCacug..................  4      1  Fm1
......................................................................ugagauUauugugaaaacug..................  48     1  Fm1
......................................................................ugagaucauugugUaaacug..................  36     1  Fm1
......................................................................ugagaucauuguUaaaacug..................  21     1  Fm1
......................................................................ugagaucauugAgaaaacug..................  15     1  Fm1
......................................................................ugagaucauugugCaaacug..................  14     1  Fm1
......................................................................ugCgaucauugugaaaacug..................  20     1  Fm1
......................................................................ugagaucauugugaaaacuU..................  321    1  Fm1
......................................................................ugagaucauugugaaaGcug..................  220    1  Fm1
......................................................................uUagaucauugugaaaacug..................  27     1  Fm1
......................................................................ugNgaucauugugaaaacug..................  1      1  Fm1
......................................................................ugagaucauugugaaUacug..................  17     1  Fm1
......................................................................ugagaucauuUugaaaacug..................  137    1  Fm1
......................................................................ugagaucauGgugaaaacuga.................  2      1  Fm1
......................................................................ugagaucauugCgaaaacuga.................  13     1  Fm1
......................................................................ugagaucauugugaaaacCga.................  9      1  Fm1
......................................................................ugagaucGuugugaaaacuga.................  77     1  Fm1
......................................................................ugagaucauugugaaaacuUa.................  13     1  Fm1
......................................................................ugagaucauuguCaaaacuga.................  5      1  Fm1
......................................................................ugNgaucauugugaaaacuga.................  1      1  Fm1
......................................................................ugagaucauugugGaaacuga.................  21     1  Fm1
......................................................................ugagaucauugugaaaaAuga.................  753    1  Fm1
......................................................................Cgagaucauugugaaaacuga.................  17     1  Fm1
......................................................................ugagaucauugugaUaacuga.................  4      1  Fm1
......................................................................ugagaucauugugaCaacuga.................  2      1  Fm1
......................................................................ugagaucCuugugaaaacuga.................  3      1  Fm1
......................................................................ugagaucauugugaaCacuga.................  2      1  Fm1
......................................................................ugagaGcauugugaaaacuga.................  2      1  Fm1
......................................................................ugagaucauugugaaaaUuga.................  450    1  Fm1
......................................................................ugagaucauugugaaaacuga.................  89737  0  Fm1
......................................................................ugUgaucauugugaaaacuga.................  14     1  Fm1
......................................................................ugagauUauugugaaaacuga.................  29     1  Fm1
......................................................................ugaUaucauugugaaaacuga.................  5      1  Fm1
......................................................................uNagaucauugugaaaacuga.................  2      1  Fm1
```

```
                                                    Star                              Mature
ugauguuggcuguaaugaccuucagacgaaacugguuuucauaaugaccuuccagauuguugugcuauucugagaucauugugaaaacugauuccguugccccuaauc
......................................................................ugagaucauCgugaaaacuga.................   20     1     Fm1
......................................................................ugagaucauugugaaaacugU.................   1923   1     Fm1
......................................................................ugagaucaCugugaaaacuga.................   5      1     Fm1
......................................................................ugagGucauugugaaaacuga.................   14     1     Fm1
......................................................................ugagaucauugugUaaacuga.................   16     1     Fm1
......................................................................ugagCucauugugaaaacuga.................   15     1     Fm1
......................................................................ugagaucauuCugaaaacuga.................   97     1     Fm1
......................................................................ugagaAcauugugaaaacuga.................   7      1     Fm1
......................................................................ugagaucauugAgaaaacuga.................   3      1     Fm1
......................................................................ugagaucauuAugaaaacuga.................   167    1     Fm1
......................................................................ugagaucauugugaaaacugC.................   53     1     Fm1
......................................................................uAagaucauugugaaaacuga.................   33     1     Fm1
......................................................................ugagauGauugugaaaacuga.................   4      1     Fm1
......................................................................ugagaNcauugugaaaacuga.................   17     1     Fm1
......................................................................uUagaucauugugaaaacuga.................   18     1     Fm1
......................................................................ugagaucauugugaaaacuCa.................   3      1     Fm1
......................................................................ugagaucauugugaaaacugG.................   4306   1     Fm1
......................................................................ugagaucauugugaaaacuAa.................   11     1     Fm1
......................................................................uCagaucauugugaaaacuga.................   6      1     Fm1
......................................................................Ngagaucauugugaaaacuga.................   45     1     Fm1
......................................................................ugagaucauugugaaaCcuga.................   130    1     Fm1
......................................................................ugagaucauugugaaUacuga.................   7      1     Fm1
......................................................................ugagaucUuugugaaaacuga.................   20     1     Fm1
......................................................................ugagaCcauugugaaaacuga.................   6      1     Fm1
......................................................................Agagaucauugugaaaacuga.................   4      1     Fm1
......................................................................ugagaucauugugaaaaGuga.................   383    1     Fm1
......................................................................ugagauAauugugaaaacuga.................   6      1     Fm1
......................................................................ugaAaucauugugaaaacuga.................   11     1     Fm1
......................................................................ugagaucauugugaGaacuga.................   10     1     Fm1
......................................................................ugagaucauuguAaaaacuga.................   12     1     Fm1
......................................................................ugagaucauugGgaaaacuga.................   2      1     Fm1
......................................................................ugagaucauugugaaaNcuga.................   1      1     Fm1
......................................................................ugagaucaAugugaaaacuga.................   7      1     Fm1
......................................................................ugCgaucauugugaaaacuga.................   8      1     Fm1
......................................................................ugagUucauugugaaaacuga.................   8      1     Fm1
......................................................................ugagaucauuguUaaaacuga.................   9      1     Fm1
......................................................................ugagaucauugugaaaGcuga.................   110    1     Fm1
......................................................................ugagaucaGugugaaaacuga.................   5      1     Fm1
......................................................................ugagaucauugugaaaUcuga.................   199    1     Fm1
......................................................................ugagaucauugugaaaacAga.................   3      1     Fm1
......................................................................ugagaucauuUugaaaacuga.................   72     1     Fm1
......................................................................ugaCaucauugugaaaacuga.................   5      1     Fm1
......................................................................ugagaucauugugaaGacuga.................   10     1     Fm1
......................................................................ugGgaucauugugaaaacuga.................   31     1     Fm1
......................................................................ugagaucauAgugaaaacuga.................   4      1     Fm1
......................................................................ugagauNauugugaaaacuga.................   2      1     Fm1
......................................................................ugagaucauugugCaaacuga.................   4      1     Fm1
......................................................................Ggagaucauugugaaaacugau................   1      1     Fm1
......................................................................ugagaucauugugGaaacugau................   17     1     Fm1
......................................................................ugaUaucauugugaaaacugau................   4      1     Fm1
......................................................................ugagaucauugugaaaaAugau................   923    1     Fm1
......................................................................ugagaucauugugaaaacuUau................   11     1     Fm1
......................................................................ugagaucauGgugaaaacugau................   4      1     Fm1
......................................................................Ngagaucauugugaaaacugau................   51     1     Fm1
......................................................................ugCgaucauugugaaaacugau................   12     1     Fm1
......................................................................ugagauAauugugaaaacugau................   9      1     Fm1
......................................................................ugagauGauugugaaaacugau................   4      1     Fm1
......................................................................ugagaCcauugugaaaacugau................   4      1     Fm1
......................................................................ugGgaucauugugaaaacugau................   36     1     Fm1
......................................................................ugagaucauugugaaaacuCau................   3      1     Fm1
......................................................................ugagaNcauugugaaaacugau................   13     1     Fm1
......................................................................ugagaucauugugaaaacCgau................   21     1     Fm1
......................................................................ugagaucauuCugaaaacugau................   93     1     Fm1
......................................................................ugaCaucauugugaaaacugau................   3      1     Fm1
......................................................................ugagaucauCgugaaaacugau................   18     1     Fm1
......................................................................ugagaucauugugCaaacugau................   9      1     Fm1
......................................................................ugagaucauugugaaaaUugau................   522    1     Fm1
......................................................................ugagaucauugugaaUacugau................   8      1     Fm1
......................................................................uAagaucauugugaaaacugau................   41     1     Fm1
......................................................................ugagaucauugugaaaacugUu................   54     1     Fm1
```

Star **Mature**

ugauguuggcuguaaugaccuucagacgaaacugguuuucauaaaugaccuuccagauuguugugcuauucugagaucauugugaaaacugauuccguugccccuaauc			
....ugagaucauugAgaaaacugau................	10	1	Fm1
....ugagaucauugugUaaacugau................	21	1	Fm1
....ugagCucauugugaaaacugau................	28	1	Fm1
....ugagaucauugugaaaGcugau................	157	1	Fm1
....ugagaucauugugaaaacugaG................	15	1	Fm1
....ugagaucauugugaaaCcugau................	166	1	Fm1
....ugagaucauAgugaaaacugau................	9	1	Fm1
....uNagaucauugugaaaacugau................	3	1	Fm1
....uUagaucauugugaaaacugau................	14	1	Fm1
....ugagaucauugugaaaaGugau................	446	1	Fm1
....ugagaucauugugaaCacugau................	1	1	Fm1
....ugagaucauugugaaaacugGu................	17	1	Fm1
....ugagaucauugugaaaacuAau................	7	1	Fm1
....ugagaucCuugugaaaacugau................	2	1	Fm1
....ugagauNauugugaaaacugau................	2	1	Fm1
....ugagaucUuugugaaaacugau................	25	1	Fm1
....ugagaucauuguAaaaacugau................	9	1	Fm1
....ugagaucauugugaaaacGgau................	1	1	Fm1
....ugagaucauugGgaaaacugau................	3	1	Fm1
....uCagaucauugugaaaacugau................	7	1	Fm1
....Agagaucauugugaaaacugau................	8	1	Fm1
....ugagaucauugugaUaacugau................	6	1	Fm1
....ugagaucauugugaaaUcugau................	170	1	Fm1
....ugagaucauugugaaaacugaA................	760	1	Fm1
....ugagauUauugugaaaacugau................	44	1	Fm1
....ugagUucauugugaaaacugau................	19	1	Fm1
....ugagaucauuUugaaaacugau................	74	1	Fm1
....ugagaucauugugaGaacugau................	13	1	Fm1
....ugagaucauugugaaaacugaC................	30	1	Fm1
....ugagaucauugugaCaacugau................	2	1	Fm1
....ugagaGcauugugaaaacugau................	1	1	Fm1
....ugagaucaAugugaaaacugau................	3	1	Fm1
....Cgagaucauugugaaaacugau................	40	1	Fm1
....ugagaucauugugaaGacugau................	18	1	Fm1
....ugagaucauugugaaaacugau................	105108	0	Fm1
....ugagaucauugugaaaacugCu................	11	1	Fm1
....ugagaucauuguUaaaacugau................	19	1	Fm1
....ugagaucauuAugaaaacugau................	211	1	Fm1
....ugUgaucauugugaaaacugau................	17	1	Fm1
....ugagGucauugugaaaacugau................	10	1	Fm1
....ugagaucGuugugaaaacugau................	75	1	Fm1
....ugagaAcauugugaaaacugau................	5	1	Fm1
....ugaAaucauugugaaaacugau................	10	1	Fm1
....ugagaucaCugugaaaacugau................	7	1	Fm1
....ugagaucauuguCaaaacugau................	5	1	Fm1
....ugagaucauugCgaaaacugau................	29	1	Fm1
....ugagaucauugugaaaacAgau................	3	1	Fm1
....ugagaucUuugugaaaacugauu...............	37	1	Fm1
....ugagaucauugugaaaacuCauu...............	7	1	Fm1
....ugGgaucauugugaaaacugauu...............	82	1	Fm1
....ugagaucauugugaaaacugaAu...............	47	1	Fm1
....ugagaucauCgugaaaacugauu...............	30	1	Fm1
....ugagaucauugugaaGacugauu...............	24	1	Fm1
....ugagaucauugugCaaacugauu...............	10	1	Fm1
....ugagaucauugugaaaaAugauu...............	1579	1	Fm1
....ugagaucauugugaaaGcugauu...............	224	1	Fm1
....ugagaGcauugugaaaacugauu...............	7	1	Fm1
....ugCgaucauugugaaaacugauu...............	26	1	Fm1
....ugagaucauuguUaaaacugauu...............	24	1	Fm1
....ugagaucauuUugaaaacugauu...............	127	1	Fm1
....ugagaucauugugaaaacCgauu...............	18	1	Fm1
....ugagaucauugugaaaacuAauu...............	21	1	Fm1
....ugagaucauuAugaaaacugauu...............	411	1	Fm1
....Ggagaucauugugaaaacugauu...............	8	1	Fm1
....uNagaucauugugaaaacugauu...............	2	1	Fm1
....ugagaucaCugugaaaacugauu...............	22	1	Fm1
....ugagaucGuugugaaaacugauu...............	346	1	Fm1
....ugagaucauugugaaaacugUuu...............	59	1	Fm1
....uUagaucauugugaaaacugauu...............	23	1	Fm1
....ugagaucCuugugaaaacugauu...............	5	1	Fm1

```
                                   Star                               Mature
ugauguuggcuguaaugaccuucagacgaaacugguuuucauaaugaccuuccagauuguugugcuauucugagaucauugugaaaacugauuccguugccccuaauc
......................................................................ugaCaucauugugaaaacugauu...............   10       1   Fm1
......................................................................uCagaucauugugaaaacugauu...............   15       1   Fm1
......................................................................ugagaucauugugaaaacugaGu...............   7        1   Fm1
......................................................................ugagaucauugugaaaacugCuu...............   11       1   Fm1
......................................................................Cgagaucauugugaaaacugauu...............   65       1   Fm1
......................................................................ugagaucauugugaGaacugauu...............   33       1   Fm1
......................................................................ugagaucauugugaaaaGugauu...............   811      1   Fm1
......................................................................ugagauAauugugaaaacugauu...............   6        1   Fm1
......................................................................ugagaucauuguAaaaacugauu...............   34       1   Fm1
......................................................................ugagaucaAugugaaaacugauu...............   13       1   Fm1
......................................................................ugagauUauugugaaaacugauu...............   71       1   Fm1
......................................................................ugagauNauugugaaaacugauu...............   11       1   Fm1
......................................................................ugagaucauugugaaaacAgauu...............   11       1   Fm1
......................................................................ugagaucauugugaaUacugauu...............   28       1   Fm1
......................................................................ugagaucauugGgaaaacugauu...............   5        1   Fm1
......................................................................ugaUaucauugugaaaacugauu...............   9        1   Fm1
......................................................................ugagaucauugugaaaUcugauu...............   322      1   Fm1
......................................................................ugagaucauugugaaaacuUauu...............   13       1   Fm1
......................................................................uAagaucauugugaaaacugauu...............   61       1   Fm1
......................................................................ugagaucauGgugaaaacugauu...............   13       1   Fm1
......................................................................ugagaucauugAgaaaacugauu...............   24       1   Fm1
......................................................................ugagaucauugugaaaacugauA...............   1483     1   Fm1
......................................................................ugagaucauuCugaaaacugauu...............   162      1   Fm1
......................................................................ugagaucauugugaaaacugaCu...............   15       1   Fm1
......................................................................ugagaucauugugUaaacugauu...............   30       1   Fm1
......................................................................ugagaucauugugaaaacugauC...............   66       1   Fm1
......................................................................ugagaucauugugaaaacugauG...............   24       1   Fm1
......................................................................ugagaucauugugaaaacGgauu...............   12       1   Fm1
......................................................................ugagaucauugugaaCacugauu...............   6        1   Fm1
......................................................................ugNgaucauugugaaaacugauu...............   1        1   Fm1
......................................................................ugagaAcauugugaaaacugauu...............   20       1   Fm1
......................................................................ugagauGauugugaaaacugauu...............   9        1   Fm1
......................................................................Ngagaucauugugaaaacugauu...............   96       1   Fm1
......................................................................ugagaucauugugaaaacugauu...............   186320   0   Fm1
......................................................................ugagaCcauugugaaaacugauu...............   23       1   Fm1
......................................................................Agagaucauugugaaaacugauu...............   19       1   Fm1
......................................................................ugagaNcauugugaaaacugauu...............   23       1   Fm1
......................................................................ugaAaucauugugaaaacugauu...............   18       1   Fm1
......................................................................ugagGucauugugaaaacugauu...............   23       1   Fm1
......................................................................ugagaucauugugGaaacugauu...............   42       1   Fm1
......................................................................ugagaucauugugaaaaNugauu...............   1        1   Fm1
......................................................................ugagaucauugugaCaacugauu...............   4        1   Fm1
......................................................................ugagaucauAgugaaaacugauu...............   7        1   Fm1
......................................................................ugagaucauugugaaaacugGuu...............   24       1   Fm1
......................................................................ugagaucaGugugaaaacugauu...............   7        1   Fm1
......................................................................ugagCucauugugaaaacugauu...............   45       1   Fm1
......................................................................ugagaucauugugaaaaUugauu...............   909      1   Fm1
......................................................................ugUgaucauugugaaaacugauu...............   32       1   Fm1
......................................................................ugagaucauugCgaaaacugauu...............   32       1   Fm1
......................................................................ugagaucauuguCaaaacugauu...............   7        1   Fm1
......................................................................ugagUucauugugaaaacugauu...............   35       1   Fm1
......................................................................ugagaucauugugaUaacugauu...............   20       1   Fm1
......................................................................ugagaucauugugaaaCcugauu...............   318      1   Fm1
......................................................................ugagaucauugugaaaacugauuG..............   88       1   Fm1
......................................................................ugagaucauugugaaaacugauuA..............   857      1   Fm1
......................................................................ugagaucauugugaaaacugauuc..............   46       0   Fm1
......................................................................ugagaucauugugaaaacugauuU..............   15122    1   Fm1
.......................................................................Uagaucauugugaaaacu...................   1        1   Fm1
.......................................................................gagaucauuAugaaaacug..................   1        1   Fm1
.......................................................................gagaucauuguUaaaacug..................   1        1   Fm1
.......................................................................gagaucauugAgaaaacug..................   1        1   Fm1
.......................................................................Aagaucauugugaaaacug..................   1        1   Fm1
.......................................................................gagaucauugugaaaacug..................   65       0   Fm1
.......................................................................Uagaucauugugaaaacug..................   1        1   Fm1
.......................................................................gagaucauugugaaaacuU..................   1        1   Fm1
.......................................................................gagaucauuUugaaaacuga.................   1        1   Fm1
.......................................................................gagaucauugugaaaacuga.................   5        0   Fm1
.......................................................................gagaucauugugaaaacugaA................   2        1   Fm1
.......................................................................gagaucauugugaaaacugau................   3        0   Fm1
.......................................................................Uagaucauugugaaaacugau................   1        1   Fm1
```

Star **Mature**

```
ugauguuggcuguaaugaccuucagacgaaacugguuuucauaaugaccuuccagauuguugugcuauucugagaucauugugaaaacugauuccguugccccuaauc
.......................................................................gagaucauugugaaaacugauu............... 20 0 Fm1
.......................................................................Uagaucauugugaaaacugauu............... 4 1 Fm1
........................................................................Ugaucauugugaaaacug.................. 1 1 Fm1
........................................................................agaucauugugaaaacug.................. 1 0 Fm1
........................................................................Ugaucauugugaaaacuga................. 3 1 Fm1
........................................................................agaucauugugaaaacuga................. 2 0 Fm1
........................................................................Ugaucauugugaaaacugau................ 2 1 Fm1
........................................................................agaucauugugaaaacugauu............... 8 0 Fm1
........................................................................Ugaucauugugaaaacugauu............... 3 1 Fm1
.........................................................................gaucauugugaaaacuga................. 2 0 Fm1

...............................cugguuuucauaaugacAuuccaga.................................................... 1 1 Fm2
...............................cugguuuucauaaugaccuuccaga.................................................... 33 0 Fm2
...................................uuuucauaaugaccuuccaga.................................................... 10 0 Fm2
...................................................................uucugagaucauugugaaa...................... 3 0 Fm2
...................................................................uucAgagaucauugugaaaa..................... 1 1 Fm2
...................................................................uucugagaucauugugaaaa..................... 5 0 Fm2
...................................................................uucugagaucauugugaaaac.................... 5 0 Fm2
...................................................................uucugGgaucauugugaaaacu................... 1 1 Fm2
...................................................................uucugagaucGuugugaaaacu................... 2 1 Fm2
...................................................................uucugagaucauugugaaaacG................... 8 1 Fm2
...................................................................uuAugagaucauugugaaaacu................... 1 1 Fm2
...................................................................uucugagaucauugugaUaacu................... 1 1 Fm2
...................................................................uucuAagaucauugugaaaacu................... 2 1 Fm2
...................................................................uucugagaucauugugaCaacu................... 3 1 Fm2
...................................................................uucugagaucauugugGaaacu................... 1 1 Fm2
...................................................................uucugagaucauugugUaaacu................... 1 1 Fm2
...................................................................uucAgagaucauugugaaaacu................... 1 1 Fm2
...................................................................uucugagaucauugugaGaacu................... 2 1 Fm2
...................................................................uucugagaucauugugaaGacu................... 1 1 Fm2
...................................................................uuGugagaucauugugaaaacu................... 1 1 Fm2
...................................................................uucugagaucauugugaaaacu................... 1133 0 Fm2
...................................................................uucugagaucaGugugaaaacu................... 1 1 Fm2
...................................................................uucugagaucUuugugaaaacu................... 1 1 Fm2
...................................................................uucugagaucauugugCaaacu................... 6 1 Fm2
...................................................................uucugNgaucauugugaaaacu................... 1 1 Fm2
...................................................................uucugagaucauugugaaaacuU.................. 19 1 Fm2
...................................................................uucugagaucauugugCaaacug.................. 1 1 Fm2
...................................................................uucugagaucauugugaaaacuA.................. 2 1 Fm2
...................................................................uucugagaucauugugaaaacug.................. 98 0 Fm2
...................................................................uuAugagaucauugugaaaacug.................. 1 1 Fm2
...................................................................uucugagaucauugugaGaacug.................. 1 1 Fm2
.....................................................................cugagaucauugugaaaacug.................. 12 0 Fm2
......................................................................ugagaucauugugaaaaU.................... 5 1 Fm2
......................................................................ugagaucauugugaaaaG.................... 95 1 Fm2
......................................................................ugagaucauugugaaaaA.................... 81 1 Fm2
......................................................................ugagaucauugugaaaac.................... 80 0 Fm2
......................................................................Ngagaucauugugaaaacu................... 2 1 Fm2
......................................................................ugagaucauugugaaaacA................... 1 1 Fm2
......................................................................ugagauUauugugaaaacu................... 2 1 Fm2
......................................................................ugagaCcauugugaaaacu................... 1 1 Fm2
......................................................................ugagaucGuugugaaaacu................... 27 1 Fm2
......................................................................ugagaAcauugugaaaacu................... 2 1 Fm2
......................................................................uCagaucauugugaaaacu................... 1 1 Fm2
......................................................................Cgagaucauugugaaaacu................... 2 1 Fm2
......................................................................ugagaucauugugaaaGcu................... 6 1 Fm2
......................................................................ugagaucauugAgaaaacu................... 1 1 Fm2
......................................................................ugagaucauugugCaaacu................... 2 1 Fm2
......................................................................ugagaucauugCgaaaacu................... 1 1 Fm2
......................................................................ugagaucauugugaaaUcu................... 11 1 Fm2
......................................................................ugagaucUuugugaaaacu................... 3 1 Fm2
......................................................................ugagaucauugugaaaaAu................... 56 1 Fm2
......................................................................ugagaucauugugaGaacu................... 1 1 Fm2
......................................................................ugagaucauugugaaaacu................... 5643 0 Fm2
......................................................................ugUgaucauugugaaaacu................... 1 1 Fm2
......................................................................ugagaucauugugaaaaGu................... 30 1 Fm2
......................................................................ugGgaucauugugaaaacu................... 1 1 Fm2
......................................................................ugagaucauugugaaaacC................... 2 1 Fm2
......................................................................ugagaucauuCugaaaacu................... 7 1 Fm2
......................................................................ugagaNcauugugaaaacu................... 2 1 Fm2
```

```
                                                  Star                              Mature
ugauguuggcuguaaugaccuucagacgaaacugguuuucauaaugaccuuccagauuguugugcuauucugagaucauugugaaaacugauuccguugccccuaauc
......................................................................ugagaucauugugaaaCcu...................    9       1    Fm2
......................................................................ugagauGauugugaaaacu...................    1       1    Fm2
......................................................................ugagUucauugugaaaacu...................    1       1    Fm2
......................................................................ugagCucauugugaaaacu...................    1       1    Fm2
......................................................................ugaAaucauugugaaaacu...................    1       1    Fm2
......................................................................ugagaucauugugaaaacG...................    57      1    Fm2
......................................................................uUagaucauugugaaaacu...................    3       1    Fm2
......................................................................ugagaucauugugaaUacu...................    1       1    Fm2
......................................................................uAagaucauugugaaaacu...................    1       1    Fm2
......................................................................ugagGucauugugaaaacu...................    1       1    Fm2
......................................................................ugagaucauuguAaaaacu...................    1       1    Fm2
......................................................................ugagaucauuUugaaaacu...................    5       1    Fm2
......................................................................ugagaucauugugaaaaUu...................    35      1    Fm2
......................................................................ugagaucauugugaaCacu...................    1       1    Fm2
......................................................................Agagaucauugugaaaacu...................    1       1    Fm2
......................................................................ugagaAcauugugaaaacug..................    15      1    Fm2
......................................................................Agagaucauugugaaaacug..................    20      1    Fm2
......................................................................ugagaucauugugaaaaUug..................    1035    1    Fm2
......................................................................ugaUaucauugugaaaacug..................    11      1    Fm2
......................................................................ugagaucauugugaaaCcug..................    369     1    Fm2
......................................................................ugagaucauugugaaUacug..................    12      1    Fm2
......................................................................ugagGucauugugaaaacug..................    37      1    Fm2
......................................................................ugagaucauugugGaaacug..................    28      1    Fm2
......................................................................ugagauUauugugaaaacug..................    66      1    Fm2
......................................................................Cgagaucauugugaaaacug..................    60      1    Fm2
......................................................................ugagauNauugugaaaacug..................    13      1    Fm2
......................................................................ugagaucauuAugaaaacug..................    388     1    Fm2
......................................................................ugagaCcauugugaaaacug..................    26      1    Fm2
......................................................................ugagaucauuUugaaaacug..................    128     1    Fm2
......................................................................ugagaucauugugaaCacug..................    6       1    Fm2
......................................................................ugUgaucauugugaaaacug..................    27      1    Fm2
......................................................................ugagaucauuguAaaaacug..................    25      1    Fm2
......................................................................ugagaucauugugaaaacug..................    211480  0    Fm2
......................................................................ugagaucauugCgaaaacug..................    24      1    Fm2
......................................................................ugagaucauugugaaGacug..................    30      1    Fm2
......................................................................ugagaucauugugaaaaAug..................    1706    1    Fm2
......................................................................Ggagaucauugugaaaacug..................    9       1    Fm2
......................................................................ugGgaucauugugaaaacug..................    46      1    Fm2
......................................................................uAagaucauugugaaaacug..................    86      1    Fm2
......................................................................ugagaNcauugugaaaacug..................    32      1    Fm2
......................................................................ugagaucaGugugaaaacug..................    19      1    Fm2
......................................................................ugagaucauugugaaaacGg..................    961     1    Fm2
......................................................................ugagaucUuugugaaaacug..................    23      1    Fm2
......................................................................ugagaucauAgugaaaacug..................    16      1    Fm2
......................................................................ugagaucGuugugaaaacug..................    41      1    Fm2
......................................................................ugagauAauugugaaaacug..................    13      1    Fm2
......................................................................ugagaucauuguCaaaacug..................    10      1    Fm2
......................................................................ugagaucauugugUaaacug..................    43      1    Fm2
......................................................................ugaAaucauugugaaaacug..................    21      1    Fm2
......................................................................ugagCucauugugaaaacug..................    69      1    Fm2
......................................................................ugagaucauugugaaaaGug..................    994     1    Fm2
......................................................................ugNgaucauugugaaaacug..................    2       1    Fm2
......................................................................uUagaucauugugaaaacug..................    49      1    Fm2
......................................................................ugagaucauCgugaaaacug..................    35      1    Fm2
......................................................................ugagaucCuugugaaaacug..................    3       1    Fm2
......................................................................ugagaucauugugaCaacug..................    2       1    Fm2
......................................................................ugagaucauugugaGaacug..................    37      1    Fm2
......................................................................ugagaGcauugugaaaacug..................    5       1    Fm2
......................................................................ugagaucauugugCaaacug..................    9       1    Fm2
......................................................................ugagaucauugugaaaacuU..................    533     1    Fm2
......................................................................ugagaucaAugugaaaacug..................    12      1    Fm2
......................................................................ugaCaucauugugaaaacug..................    17      1    Fm2
......................................................................ugagaucaCugugaaaacug..................    18      1    Fm2
......................................................................ugagaucauuguUaaaacug..................    16      1    Fm2
......................................................................ugagaucauugGgaaaacug..................    9       1    Fm2
......................................................................ugagaucauugugaUaacug..................    9       1    Fm2
......................................................................ugagaucauugAgaaaacug..................    25      1    Fm2
......................................................................ugagaucauugugaaaGcug..................    255     1    Fm2
......................................................................uNagaucauugugaaaacug..................    1       1    Fm2
......................................................................Ngagaucauugugaaaacug..................    108     1    Fm2
```

Star Mature

```
ugauguuggcuguaaugaccuucagacgaaacugguuuucauaaugaccuuccagauuguugugcuauucugagaucauugugaaaacugauuccguugccccuaauc
......................................................................ugagaucauugugaaaUcug..................  380     1  Fm2
......................................................................ugagaucauugugaaaacCg..................  27      1  Fm2
......................................................................ugagaucauugugaaaacuC..................  34      1  Fm2
......................................................................ugagaucauGgugaaaacug..................  8       1  Fm2
......................................................................ugagauGauugugaaaacug..................  6       1  Fm2
......................................................................ugagaucauuCugaaaacug..................  196     1  Fm2
......................................................................ugagaucauugugaaaacuA..................  311     1  Fm2
......................................................................ugCgaucauugugaaaacug..................  8       1  Fm2
......................................................................uCagaucauugugaaaacug..................  18      1  Fm2
......................................................................ugagUucauugugaaaacug..................  27      1  Fm2
......................................................................ugagaucauugugaaaacAg..................  5       1  Fm2
......................................................................ugagauAauugugaaaacuga.................  7       1  Fm2
......................................................................ugagaucaGugugaaaacuga.................  5       1  Fm2
......................................................................ugagGucauugugaaaacuga.................  16      1  Fm2
......................................................................ugagaucauugugaaaacGga.................  4       1  Fm2
......................................................................ugaAaucauugugaaaacuga.................  24      1  Fm2
......................................................................ugagaucauugugCaaacuga.................  6       1  Fm2
......................................................................ugagaucauugugaaaacuga.................  113491  0  Fm2
......................................................................ugagaucauugugUaaacuga.................  6       1  Fm2
......................................................................ugagaucauugugaaaaGuga.................  494     1  Fm2
......................................................................ugagaCcauugugaaaacuga.................  7       1  Fm2
......................................................................ugagaucauAgugaaaacuga.................  6       1  Fm2
......................................................................ugaUaucauugugaaaacuga.................  15      1  Fm2
......................................................................ugagaucauugugaaaacAga.................  9       1  Fm2
......................................................................ugagauNauugugaaaacuga.................  5       1  Fm2
......................................................................ugagaucauugCgaaaacuga.................  16      1  Fm2
......................................................................ugagaGcauugugaaaacuga.................  3       1  Fm2
......................................................................ugagaucCuugugaaaacuga.................  3       1  Fm2
......................................................................ugagauGauugugaaaacuga.................  7       1  Fm2
......................................................................Cgagaucauugugaaaacuga.................  23      1  Fm2
......................................................................ugagaucauugugaGaacuga.................  18      1  Fm2
......................................................................uNagaucauugugaaaacuga.................  2       1  Fm2
......................................................................ugagaucauugugaaaGcuga.................  129     1  Fm2
......................................................................Ngagaucauugugaaaacuga.................  47      1  Fm2
......................................................................ugCgaucauugugaaaacuga.................  6       1  Fm2
......................................................................ugagaucauugugaaaacugU.................  1821    1  Fm2
......................................................................ugagaucauugugaaGacuga.................  19      1  Fm2
......................................................................ugagaucUuugugaaaacuga.................  21      1  Fm2
......................................................................ugagaucauugugaUaacuga.................  7       1  Fm2
......................................................................ugagaucauugugaaaacCga.................  13      1  Fm2
......................................................................ugagaucauugAgaaaacuga.................  8       1  Fm2
......................................................................ugagaucauGgugaaaacuga.................  8       1  Fm2
......................................................................ugaCaucauugugaaaacuga.................  8       1  Fm2
......................................................................Agagaucauugugaaaacuga.................  10      1  Fm2
......................................................................ugagaucauugugaaaaUuga.................  529     1  Fm2
......................................................................ugagaucauuguAaaaacuga.................  14      1  Fm2
......................................................................ugUgaucauugugaaaacuga.................  13      1  Fm2
......................................................................ugagaucauugugaaaCcuga.................  192     1  Fm2
......................................................................ugagaucauugugaaCacuga.................  2       1  Fm2
......................................................................ugagaucauuAugaaaacuga.................  226     1  Fm2
......................................................................ugagaucauugugaaaNcuga.................  1       1  Fm2
......................................................................ugagaucauugugaaUacuga.................  8       1  Fm2
......................................................................ugagaucauugugaaaacugC.................  7       1  Fm2
......................................................................ugagCucauugugaaaacuga.................  38      1  Fm2
......................................................................ugagaucauugugaaaacuCa.................  2       1  Fm2
......................................................................ugagaucaCugugaaaacuga.................  19      1  Fm2
......................................................................ugagaucauugugaaaacuAa.................  14      1  Fm2
......................................................................ugagaucauugugaaaUcuga.................  218     1  Fm2
......................................................................ugNgaucauugugaaaacuga.................  1       1  Fm2
......................................................................ugagaucauugugaaaacuUa.................  35      1  Fm2
......................................................................ugGgaucauugugaaaacuga.................  32      1  Fm2
......................................................................ugagaAcauugugaaaacuga.................  9       1  Fm2
......................................................................ugagaucauugugGaaacuga.................  15      1  Fm2
......................................................................uAagaucauugugaaaacuga.................  56      1  Fm2
......................................................................uCagaucauugugaaaacuga.................  8       1  Fm2
......................................................................ugagauUauugugaaaacuga.................  29      1  Fm2
......................................................................ugagaucaAugugaaaacuga.................  9       1  Fm2
......................................................................ugagaNcauugugaaaacuga.................  16      1  Fm2
......................................................................ugagaucauugugaCaacuga.................  1       1  Fm2
......................................................................ugagaucauuCugaaaacuga.................  97      1  Fm2
```

Star Mature

```
ugauguuggcuguaaugaccuucagacgaaacugguuuucauaaugaccuuccagauuguugugcuauucugagaucauugugaaaacugauuccguugccccuaauc
......................................................................uUagaucauugugaaaacuga.................	29	1	Fm2
......................................................................ugagaucGuugugaaaacuga.................	105	1	Fm2
......................................................................ugagaucauCgugaaaacuga.................	46	1	Fm2
......................................................................ugagaucauuUugaaaacuga.................	67	1	Fm2
......................................................................ugagaucauuguCaaaacuga.................	8	1	Fm2
......................................................................ugagaucauuguUaaaacuga.................	9	1	Fm2
......................................................................ugagaucauugGgaaaacuga.................	9	1	Fm2
......................................................................ugagaucauugugaaaacugG.................	5613	1	Fm2
......................................................................ugagaucauugugaaaaAuga.................	970	1	Fm2
......................................................................ugagUucauugugaaaacuga.................	26	1	Fm2
......................................................................ugagaucGuugugaaaacugau................	50	1	Fm2
......................................................................ugagaucauugugaaGacugau................	10	1	Fm2
......................................................................ugagaucauugugaaaacugaC................	22	1	Fm2
......................................................................ugaUaucauugugaaaacugau................	5	1	Fm2
......................................................................ugagaucauuguAaaaacugau................	9	1	Fm2
......................................................................ugagaucauugugaaUacugau................	16	1	Fm2
......................................................................ugagaGcauugugaaaacugau................	7	1	Fm2
......................................................................ugagaNcauugugaaaacugau................	16	1	Fm2
......................................................................ugagaucauugugaaaaAugau................	797	1	Fm2
......................................................................ugagaucaCugugaaaacugau................	9	1	Fm2
......................................................................ugagaucauuguCaaacugau................	4	1	Fm2
......................................................................ugagaAcauugugaaaacugau................	10	1	Fm2
......................................................................Agagaucauugugaaaacugau................	7	1	Fm2
......................................................................ugagaucauugugaaaGcugau................	150	1	Fm2
......................................................................ugagaucauugugUaaacugau................	16	1	Fm2
......................................................................ugaCaucauugugaaaacugau................	4	1	Fm2
......................................................................ugagaucauugugaGaacugau................	9	1	Fm2
......................................................................ugNgaucauugugaaaacugau................	1	1	Fm2
......................................................................ugagGucauugugaaaacugau................	15	1	Fm2
......................................................................ugagaucauugugaaaaGugau................	429	1	Fm2
......................................................................Cgagaucauugugaaaacugau................	43	1	Fm2
......................................................................ugagaucCuugugaaaacugau................	2	1	Fm2
......................................................................ugagaucauCgugaaaacugau................	16	1	Fm2
......................................................................ugagaucauugugCaaacugau................	7	1	Fm2
......................................................................ugagaucauugugaaaacAgau................	3	1	Fm2
......................................................................Ngagaucauugugaaaacugau................	45	1	Fm2
......................................................................ugagaucauugugaaaacugGu................	4	1	Fm2
......................................................................ugagaucaGugugaaaacugau................	1	1	Fm2
......................................................................ugagaucauugAgaaaacugau................	15	1	Fm2
......................................................................ugCgaucauugugaaaacugau................	9	1	Fm2
......................................................................ugagaucauugugaaaacugUu................	12	1	Fm2
......................................................................ugagaucauugugaaaacuCau................	6	1	Fm2
......................................................................ugagaucauugugaaaCcugau................	187	1	Fm2
......................................................................ugagaucauugugaaaacugau................	101226	0	Fm2
......................................................................ugagaucauugugaaaacugaA................	585	1	Fm2
......................................................................ugagaucauuAugaaaacugau................	206	1	Fm2
......................................................................ugagaucauugugaCaacugau................	1	1	Fm2
......................................................................ugGgaucauugugaaaacugau................	29	1	Fm2
......................................................................ugagaucauugGgaaaacugau................	6	1	Fm2
......................................................................ugagaucauugugaaaacugaG................	6	1	Fm2
......................................................................ugagauUauugugaaaacugau................	29	1	Fm2
......................................................................ugagaucauugugaaaacCgau................	7	1	Fm2
......................................................................ugagaucaAugugaaaacugau................	4	1	Fm2
......................................................................ugagaucauugCgaaaacugau................	21	1	Fm2
......................................................................ugagaucauugugaUaacugau................	15	1	Fm2
......................................................................ugagaucauugugGaaacugau................	12	1	Fm2
......................................................................ugagCucauugugaaaacugau................	30	1	Fm2
......................................................................uCagaucauugugaaaacugau................	12	1	Fm2
......................................................................ugagaucauuUugaaaacugau................	72	1	Fm2
......................................................................ugaAaucauugugaaaacugau................	19	1	Fm2
......................................................................ugagUucauugugaaaacugau................	14	1	Fm2
......................................................................ugagaucauugugaaaacuUau................	26	1	Fm2
......................................................................ugagaucauugugaaaaUugau................	503	1	Fm2
......................................................................ugagaucauAgugaaaacugau................	9	1	Fm2
......................................................................ugagauNauugugaaaacugau................	2	1	Fm2
......................................................................ugagaucauugugaaaacuAau................	9	1	Fm2
......................................................................ugagauGauugugaaaacugau................	2	1	Fm2
......................................................................ugagaucauugugaaaUcugau................	162	1	Fm2
......................................................................ugUgaucauugugaaaacugau................	12	1	Fm2
......................................................................ugagaucUuugugaaaacugau................	20	1	Fm2
```

```
                                   Star                                Mature
ugauguuggcuguaaugaccuucagacgaaacugguuuucauaaugaccuuccagauuguugugcuauucugagaucauugugaaaacugauuccguugccccuaauc
......................................................................ugagaucauugugaaaacugCu................    1       1    Fm2
......................................................................ugagaucauGgugaaaacugau................    2       1    Fm2
......................................................................ugagaucauugugaaaacGgau................    1       1    Fm2
......................................................................uUagaucauugugaaaacugau................    20      1    Fm2
......................................................................ugagaucauuCugaaaacugau................    93      1    Fm2
......................................................................ugagauAauugugaaaacugau................    2       1    Fm2
......................................................................ugagaucauuguUaaaacugau................    11      1    Fm2
......................................................................uNagaucauugugaaaacugau................    1       1    Fm2
......................................................................uAagaucauugugaaaacugau................    36      1    Fm2
......................................................................ugagaCcauugugaaaacugau................    18      1    Fm2
......................................................................ugagaucauCgugaaaacugauu...............    22      1    Fm2
......................................................................ugagaucauugugaaCacugauu...............    5       1    Fm2
......................................................................ugagaucauugugaaUacugauu...............    17      1    Fm2
......................................................................ugagaucauAgugaaaacugauu...............    10      1    Fm2
......................................................................uAagaucauugugaaaacugauu...............    63      1    Fm2
......................................................................ugagaCcauugugaaaacugauu...............    10      1    Fm2
......................................................................uCagaucauugugaaaacugauu...............    10      1    Fm2
......................................................................ugagUucauugugaaaacugauu...............    32      1    Fm2
......................................................................ugaAaucauugugaaaacugauu...............    19      1    Fm2
......................................................................ugagaucauugugaaaaUugauu...............    705     1    Fm2
......................................................................ugagaucauugugaaaacugaGu...............    2       1    Fm2
......................................................................ugagaucauugugaaaacugauC...............    21      1    Fm2
......................................................................ugagaucauugugaaaacugauA...............    1569    1    Fm2
......................................................................ugagaAcauugugaaaacugauu...............    25      1    Fm2
......................................................................Ggagaucauugugaaaacugauu...............    3       1    Fm2
......................................................................uNagaucauugugaaaacugauu...............    1       1    Fm2
......................................................................ugagaNcauugugaaaacugauu...............    23      1    Fm2
......................................................................ugCgaucauugugaaaacugauu...............    13      1    Fm2
......................................................................Agagaucauugugaaaacugauu...............    16      1    Fm2
......................................................................ugagaucauugGgaaaacugauu...............    4       1    Fm2
......................................................................ugGgaucauugugaaaacugauu...............    42      1    Fm2
......................................................................ugagaucauugugaaaacugCuu...............    5       1    Fm2
......................................................................ugagaucauuCugaaaacugauu...............    162     1    Fm2
......................................................................ugagaucauugugaaaacugGuu...............    7       1    Fm2
......................................................................ugagGucauugugaaaacugauu...............    21      1    Fm2
......................................................................ugagaucauugugaaaacuUauu...............    27      1    Fm2
......................................................................ugagaucauugugaCaacugauu...............    3       1    Fm2
......................................................................ugagaucauugugCaaacugauu...............    9       1    Fm2
......................................................................Ngagaucauugugaaaacugauu...............    72      1    Fm2
......................................................................ugagaucauugugaaaNcugauu...............    1       1    Fm2
......................................................................ugagauNauugugaaaacugauu...............    8       1    Fm2
......................................................................ugagaucauuUugaaaacugauu...............    100     1    Fm2
......................................................................ugagaucauugugaaaacuCauu...............    9       1    Fm2
......................................................................ugagaucauugugUaaacugauu...............    38      1    Fm2
......................................................................ugagaucauuguAaaaacugauu...............    15      1    Fm2
......................................................................ugagaucauugugaaaacuAauu...............    7       1    Fm2
......................................................................ugaUaucauugugaaaacugauu...............    7       1    Fm2
......................................................................ugagaucauugugaaaUcugauu...............    319     1    Fm2
......................................................................ugagaucauugugaaaacAgauu...............    6       1    Fm2
......................................................................ugagaucauugugaaaacugUuu...............    14      1    Fm2
......................................................................ugagaucauugugaaaacugaCu...............    16      1    Fm2
......................................................................uUagaucauugugaaaacugauu...............    36      1    Fm2
......................................................................ugagaucauugugaaaacugauG...............    27      1    Fm2
......................................................................ugagCucauugugaaaacugauu...............    51      1    Fm2
......................................................................ugagaucauugCgaaaacugauu...............    17      1    Fm2
......................................................................ugaCaucauugugaaaacugauu...............    6       1    Fm2
......................................................................ugagaucauuAugaaaacugauu...............    290     1    Fm2
......................................................................ugagaucauGgugaaaacugauu...............    4       1    Fm2
......................................................................ugagauGauugugaaaacugauu...............    8       1    Fm2
......................................................................ugagaucaAugugaaaacugauu...............    9       1    Fm2
......................................................................ugagaucaGugugaaaacugauu...............    9       1    Fm2
......................................................................ugagaucauugugaaaacugauu...............    143811  0    Fm2
......................................................................ugagauUauugugaaaacugauu...............    98      1    Fm2
......................................................................ugagaucauugugaGaacugauu...............    27      1    Fm2
......................................................................ugagaGcauugugaaaacugauu...............    6       1    Fm2
......................................................................ugagaucauugAgaaaacugauu...............    9       1    Fm2
......................................................................ugagaucaCugugaaaacugauu...............    22      1    Fm2
......................................................................ugUgaucauugugaaaacugauu...............    27      1    Fm2
......................................................................ugagaucauugugaaaaAugauu...............    1217    1    Fm2
......................................................................ugagaucauugugGaaacugauu...............    23      1    Fm2
```

```
                                   Star                              Mature
ugauguuggcuguaaugaccuucagacgaaacugguuuucauaaugaccuuccagauuguugugcuauucugagaucauugugaaaacugauuccguugccccuaauc
......................................................................ugagaucauugugaaaCcugauu...............  248    1    Fm2
......................................................................ugagaucGuugugaaaacugauu...............  281    1    Fm2
......................................................................ugagaucauugugaaaGcugauu...............  178    1    Fm2
......................................................................ugagaucauuguCaaaacugauu...............  7      1    Fm2
......................................................................ugagaucauugugaaaacCgauu...............  21     1    Fm2
......................................................................ugagaucauugugaaaacGgauu...............  4      1    Fm2
......................................................................ugagaucauugugaaaaGugauu...............  627    1    Fm2
......................................................................ugagaucUuugugaaaacugauu...............  24     1    Fm2
......................................................................ugagaucauugugaaaacugaAu...............  31     1    Fm2
......................................................................Cgagaucauugugaaaacugauu...............  32     1    Fm2
......................................................................ugagaucCuugugaaaacugauu...............  2      1    Fm2
......................................................................ugagauAauugugaaaacugauu...............  6      1    Fm2
......................................................................ugagaucauuguUaaaacugauu...............  12     1    Fm2
......................................................................ugagaucauugugaaGacugauu...............  21     1    Fm2
......................................................................ugagaucauugugaUaacugauu...............  8      1    Fm2
......................................................................ugagaucauugugaaaacugauuG..............  61     1    Fm2
......................................................................ugagaucauugugaaaacugauuA..............  790    1    Fm2
......................................................................ugagaucauugugaaaacugauuU..............  13895  1    Fm2
......................................................................ugagaucauugugaaaacugauuc..............  59     0    Fm2
......................................................................ugagaucauugugaaaacugauucU.............  7      1    Fm2
.......................................................................gagaucauugCgaaaacug..................  1      1    Fm2
.......................................................................gagaucauugugaaaacGg..................  1      1    Fm2
.......................................................................gagaucauugugaaaacuU..................  1      1    Fm2
.......................................................................Uagaucauugugaaaacug..................  7      1    Fm2
.......................................................................gagaucauugugaaaacug..................  43     0    Fm2
.......................................................................gagaucauugugaaaaAug..................  1      1    Fm2
.......................................................................gagaucauugugaaaacuga.................  5      0    Fm2
.......................................................................Uagaucauugugaaaacuga.................  2      1    Fm2
.......................................................................Uagaucauugugaaaacugau................  5      1    Fm2
.......................................................................gagaucauugugaaaacugau................  3      0    Fm2
.......................................................................gagaucauugugCaaacugauu...............  1      1    Fm2
.......................................................................Uagaucauugugaaaacugauu...............  3      1    Fm2
........................................................................Ugaucauugugaaaacugau................  1      1    Fm2
........................................................................Ugaucauugugaaaacugauu...............  4      1    Fm2
........................................................................agaucauugugaaaacugauu...............  2      0    Fm2
```
